# Assessing the effects of methylphenidate in proliferation and Wnt activity of neuronal stem cells from attention deficit/hyperactivity disorder patients

**DOI:** 10.1007/s00702-025-02988-y

**Published:** 2025-07-28

**Authors:** Cristine Marie Yde Ohki, Natalie Monet Walter, Lukasz Smigielski, Audrey Bender, Michelle Rickli, Susanne Walitza, Edna Grünblatt

**Affiliations:** 1 Department of Child and Adolescent Psychiatry and Psychotherapy, Translational Molecular Psychiatry, University Hospital of Psychiatry Zurich, University of Zurich, Wagistrasse 12, 8952 Schlieren, Switzerland; 2 Neuroscience Center Zurich, University of Zurich and the ETH Zurich, Winterthurerstrasse 11, 8057 Zurich, Switzerland; 3 Zurich Center for Integrative Human Physiology, University of Zurich, Winterthurerstrasse 11, 8057 Zurich, Switzerland

**Keywords:** ADHD, Alzheimer’s disease, Anxiety, Autism spectrum disorder, β-catenin, Bipolar disorder, GSK3β, iPSC, LRP6, Major depressive disorder, Methylphenidate, Neural stem cells, Obsessive–compulsive disorder, Polygenic risk score, Proliferation, Protein expression, Real time impedance cell analysis, Reporter assays, RTCA, Wnt signaling, WST-1, xCELLigence

## Abstract

As the most common neurodevelopmental and mental disorder around the world, attention-deficit/hyperactivity disorder (ADHD) affects primarily children and adolescents. Both genetic (polygenicity) and environmental variables interplay in the etiology of this disorder. The Wnt signaling pathway, which regulates proliferation and differentiation during neurodevelopment, has been implicated in ADHD. Clinically, individuals with ADHD may exhibit delays in structural and functional brain development. Available evidence suggests that methylphenidate (MPH) treatment can potentially improve these delays. However, the molecular and cellular mechanisms underlying ADHD and the therapeutic targets of MPH are not yet completely elucidated. In a pilot investigation, the proliferation of neural stem cells (NSCs) derived from induced pluripotent stem cells (iPSCs) was significantly lowered in ADHD male patients. Yet, we did not observe any variations in proliferation rates during the iPSC stage. To extend the earlier results, we increased the sample size to include females, explored whether MPH may improve NSC proliferation in ADHD and clarified the role of the Wnt pathway. To do so, iPSC and NSC proliferation from five ADHD patients and five controls was assessed. The results corroborated our previous findings of decreased proliferation in ADHD NSCs. Conversely, ADHD NSC proliferation was modestly regulated by MPH treatment at 10 nM, which also showed modulatory effects on Wnt signaling in this group. Interestingly, no increase in proliferation was seen when DKK1 blocked Wnt signaling before MPH treatment. These findings suggest MPH regulates the canonical Wnt pathway and may partially explain ADHD-related neurodevelopmental abnormalities and MPH-specific benefits.

**Supplementary Information:**

The online version contains supplementary material available at 10.1007/s00702-025-02988-y.

## Introduction

Methylphenidate (MPH), a psychostimulant, is commonly employed to treat attention-deficit/hyperactivity disorder (ADHD), a multifactorial neurodevelopmental disorder that is often characterized by delays in brain maturation of up to 4 years compared to controls (Nakao et al. 2011). Structural delays include decreased cortical thickness (Hoogman et al. 2017, 2019) due to common gray and white matter reductions in regions highly implicated in ADHD-related cognitive functions, such as attention (Rubia et al. 2009, 2011) and motor response inhibition (Rubia et al. 1999; Durston et al. 2003). These include the basal ganglia (Ellison-Wright et al. 2008; Konrad and Eickhoff 2010) and prefrontal cortex (Francx et al. 2016).

MPH may normalize gray and white matter volumes in ADHD compared to unmedicated patients (Schweren et al. 2013). In addition to structural ameliorations, MPH may improve functional delays in ADHD brain networks (Schweren et al. 2013). Among other molecular mechanisms, MPH blocks dopamine and norepinephrine transporters (DAT and NET, respectively) (Kuczenski and Segal 1997; Gatley et al. 1996; Faraone 2018). MPH has one of the largest effect sizes in children and adolescent psychiatry (Cortese et al. 2018; Correll et al. 2021), reducing hyperactivity and impulsivity and improving attention in ADHD patients (Whalen and Henker 1991). This constitutes a paradoxical impact as it seems counterintuitive given its psychostimulant properties (Robbins and Sahakian 1979; Green and Warshauer 1981). *In vivo*, MPH-induced behavioral benefits persisted in DAT-knockout mice (Huang and Huang 2012), suggesting that MPH may have additional means of action (Faraone 2018).

ADHD has been linked to the Wnt signaling pathway (Yde Ohki et al. 2020; Grünblatt et al. 2019). In a review paper, MacDonald et al. detailed how extracellular Wnt ligands like Wnt3a, Wnt5a, and Wnt7a activate transmembrane receptors LRP5/6 (LDL receptor-related protein 5/6) and Frizzled (FZD) triggering the Wnt cascade. Dickkopf-related protein 1 (DKK1), a well-known Wnt antagonist, can internalize LRP5/6 receptors through Kremen-mediated endocytosis (Mao et al. 2002) or compete with extracellular Wnt ligands for LRP6 (Bourhis et al. 2010). In the absence of Wnt agonists or the presence of antagonists, GSK3 $\upbeta$, a key component of the “destruction complex”, phosphorylates and degrades $\upbeta$-catenin. GSK3 $\upbeta$ becomes inactive when phosphorylated at Serine-9 (S9), preventing it from phosphorylating $\upbeta$-catenin for proteasomal degradation. When Wnt ligands activate LRP5/6 and FZD receptors in the extracellular milieu, a cascade is activated, inhibiting the destruction complex and allowing β-catenin to accumulate and translocate into the nucleus. This activation of Wnt target genes is aided by the TCF/LEF (T cell factor/lymphoid enhancer factor) family of transcription factors (MacDonald et al. 2009). These target genes may be closely associated with essential neurodevelopmental cellular processes like proliferation, cell fate specification, and differentiation, which may be modulated differentially in distinct cell types (Sethi and Vidal-Puig 2010).

Induced pluripotent stem cells (iPSCs) can preserve the genetic background of somatic cells and allow the study of patient-specific molecular phenotypes in functional living neural cells (Yde Ohki et al. 2022). Our preliminary data revealed that male ADHD neural stem cells (NSCs) proliferate more slowly than controls, whereas iPSCs (early developmental stage) proliferate similarly between groups (Yde Ohki et al. 2023a). Our paper showed that ADHD NSCs have higher basal Wnt activity than controls in terms of protein expression and function (Walter et al. 2024). The current study examined the proliferation in a larger number of cell lines, including both sexes. We also investigated whether MPH can reverse ADHD NSC proliferation via a Wnt-dependent mechanism.

## Material and methods

### Recruitment of participants

According to Yde Ohki et al. (2023a), the Department of Child and Adolescent Psychiatry and Psychotherapy (KJPP) of the University of Zurich (UZH) recruited ADHD patients aged 6 − 18 who clinically respond to MPH treatment with no comorbidities, and matched healthy controls. The inclusion and exclusion criteria have been outlined in previous publications (Grossmann et al. 2021; Yde Ohki et al. 2021, 2023c, 2024) and a recently published paper (Walter et al. 2024).

As part of the recruitment process, ADHD patients and controls were evaluated using the Child Behavior Checklist (CBCL) from parents (Achenbach and Edelbrock 1991) and Conners-3-Rating Scales (Conners et al. 2011) from patients, teachers and parents. Controls were required to have lower Conners’ T-values (< 60) in hyperactivity/impulsivity and inattention whereas ADHD patients were required to score at least 65 in each symptomatology scale in at least one of the Conners’ questionnaires (Yde Ohki et al. 2023c, 2021; Grossmann et al. 2021).

As previously reported (Grossmann et al. 2021; Yde Ohki et al. 2021, 2023c), salivary DNA samples from patients and controls were genotyped after recruitment. Individual Polygenic Risk Scores (PRS) were calculated using a clumping/thresholding method for *p* = 0.05 in PLINK (Walter et al. 2024) to quantify genetic predisposition to ADHD (Demontis et al. 2023) and other neuropsychiatric disorders (Alzheimer’s disease (AD) (Wightman et al. 2021), Autism Spectrum Disorder (ASD) (Grove et al. 2019), bipolar disorder (BD) (Mullins et al. 2021), major depressive disorder (MDD) (Wray et al. 2018), obsessive–compulsive disorder (OCD) (International Obsessive Compulsive Disorder Foundation Genetics Collaborative (IOCDF-GC) and OCD Collaborative Genetics Association Studies (OCGAS) 2018). PRS for anxiety (AN) was calculated using the summary statistics provided by Prof. Dr. Thalia Eley and Gerome Breen from Purves et al. (2020). PRSet was used to calculate ADHD (Choi et al. 2023) pathway-PRS for the Wnt pathway (Molecular Signatures Database version v2023.2.Hs; source code: hsa04310). Baldness PRS (Hagenaars et al. 2017) was computed and analyzed as a negative PRS control (Walter et al. 2024). Supplementary Table 1 lists the study subjects and their demographic and clinical characteristics including CBCL and Conners-3 Rating Scales.

As previously reported (Yde Ohki et al. 2021, 2023a, 2023b, 2023c; Grossmann et al. 2021), the Cantonal Ethics Committee (BASEC-Nr.-2016–00101 & BASEC-Nr.-201700825) approved this project, which followed the latest version of the Declaration of Helsinki. All participants and/or parents signed the study consent form.

### Generation and culture of iPSCs and NSCs

IPSCs from plucked hair-derived keratinocytes or peripheral blood mononuclear cells (PBMCs) from ADHD patients and healthy controls were generated through Sendai virus transduction and quality controlled (QCed) as reported in previous publications (Grossmann et al. 2021; Yde Ohki et al. 2021, 2023c, 2024). These cell lines underwent extensive QC that included verification of genomic integrity using Single Nucleotide Polymorphism (SNP) arrays, mycoplasma testing, Sendai virus detection, Copy Number Variation (CNV) analysis, embryoid body (EB) formation, and assessment of gene and protein expression of pluripotency markers (Grossmann et al. 2021; Yde Ohki et al. 2021, 2023c, 2024).

NSCs were generated using the Gibco PSC Neural Induction protocol as previously described (Yde Ohki et al. 2023b). The culture of NSCs and their QC in terms of gene and protein expression analysis through qRT-PCR and immunocytochemistry, respectively, were performed as described in our previously published papers (Yde Ohki et al. 2023a; Walter et al. 2024). The NSCs utilized in this study were positive for classical NSC markers (*i.e.*, SOX2, TUJ1, NESTIN, and PAX6) (Yde Ohki et al. 2023a; Walter et al. 2024) (Supplementary Fig. 1). Supplementary Table 2 outlines the sources of QC for these cell lines as documented in the literature and/or in the current study.

### xCELLigence and WST-1 assays for iPSC and NSC proliferation

Human iPSCs and NSCs that had successfully undergone QC were submitted to the real-time impedance cell analysis, xCELLigence, and the colorimetric assay WST-1, as described in Yde Ohki et al. (2023a).

Briefly, on day 0, 25,000 iPSCs in Essential 8 Flex (Gibco™) were seeded per well in an E96 Plate (OLS^®^ BIO) or in regular 96-well plates (Sarstedt) for xCELLigence and WST-1, respectively. These plates were coated with Vitronectin (Gibco™) at 15 μg/mL and 5 μg/mL, respectively. Similarly, 15,000 NSCs were seeded onto E96-well plates (Agilent) or in regular 96-well plates (Sarstedt) coated with Matrigel (Corning^®^ Matrigel^®^ hESC-Qualified Matrix) diluted 1:100 in DMEM/F12 (Gibco™) in Neural Expansion Media (NEM; PSC Neural Induction Medium, Gibco™) for xCELLigence and WST-1 assays, respectively. We determined the proliferation rates from xCELLigence by fitting cell index (CI) curves to Malthusian proliferation models using R Statistical Software v. 4.1.2. (Yde Ohki et al. 2023a).

In xCELLigence, the first measurement occurs in the first experimental minute to detect the impedance in the wells containing only media. For baseline experiments, xCELLigence CI was measured for 2 h every 10 min, followed by measurements every hour throughout a 24-sweep cycle. Next, the xCELLigence Real-Time Cell Analysis (RTCA) station recorded measurements every hour throughout two 48-h sweep cycles. This schedule allowed for media refreshment during sweep intervals. The experiment finished after 120 h. The slope of the impedance curves from 24 h post-seeding until their maximum CI was determined. For WST-1, the same number of cells was seeded onto wells of 96well plates, and WST-1 tetrazolium salt (Sigma) was added to the wells at the timepoints of 24 h, 42 h, 48 h, 66 h, 72 h, 90 h, 96 h, and 114 h post-seeding. Absorbance measurements at 440 nm took place 4 h later using the Mithras2 LB 943 Multimode Reader (Berthold Technologies) and adopting a reference wavelength of 630 nm, as previously reported (Yde Ohki et al. 2023a). Absorbance curves were log2-transformed for linearization, and proliferation rates were calculated as the slopes after fitting the curve from 24 h to the maximum absorbance in linear regression models (Yde Ohki et al. 2023a).

Regarding data analysis, data from xCELLigence wells per replicate were cleaned using the Interquartile Range (IQR) method, whereas WST-1 wells per timepoint per replicate were cleaned using a z-score method, consistent with our previous publication (Yde Ohki et al. 2023a). Replicates were averaged per cell line and represented as dots in each graph.

Each investigated group included four males and one female (including 2 iPSC clones per person) (see Supplementary Table 1). The average of two technical replicate trials for each cell line was used for all analyses. For iPSCs, WST-1 assays involved conducting a minimum of two technical replicates per cell line, which were then averaged.

### MPH treatment of NSCs for subsequent evaluation of proliferation

The cells were chronically treated with MPH hydrochloride (Lipomed AG, MPH-1043-HC) every 24 h at 10 nM and 100 nM for 5 experimental days to measure proliferation using xCELLigence and WST-1. Cell index (CI) values were assessed every 10 min for 2 h followed by every hour for 24 h, as in baseline experiments. Four 24 h sweep cycles were then performed to ensure daily treatments.

Here, the slope of impedance curves in xCELLigence from the moment that MPH (or vehicle) was added for the first time to the cultures (24 h and 24.5 h post-seeding for experiments with MPH and DKK1, respectively) until its maximum CI was calculated as proliferation rates.

On days 1 and 3, the NSCs were completely refreshed with NEM and MPH. On days 2 and 4 after seeding, only MPH or vehicle (water) was added to the wells. Figure 2A depicts the experimental design of these assays.

The doses of 10 and 100 nM MPH were selected based on mean serum MPH concentrations of approximately 17.6 ng/mL and 16.4 ng/mL (ca. 70 nM) observed in children at the pharmacological peak of 2 h post-administration of 0.9 and 1.1 mg/kg (Preiskorn et al. 2018), which are considered therapeutic doses (Wargin et al. 1983). Considering that interindividual differences in pharmacodynamics and pharmacokinetics may occur and that serum/plasma levels of MPH might not represent the concentrations found in the CNS (since they may get previously metabolized while a portion of the drug might not cross the blood–brain barrier due to several factors (e.g., lipophilicity) (Summerfield et al. 2007), 10 nM and 100 nM may better represent the concentrations that reach the CNS.

### DKK1 treatment of NSCs with and without MPH

To investigate the Wnt signaling system’s effect on MPH proliferation, DKK1 was administered before MPH using xCELLigence (Fig. 3A). To prepare an intermediate solution at 600 ng/mL, a stock solution of DKK1 at 10,000 ng/mL in water with 1% Bovine Serum Albumin (BSA) was freshly diluted in NEM. The wells’ media were replaced with fresh NEM and DKK1 on days 1 and 3 post-seeding, whereas DKK1 was added only on days 2 and 4 (final concentration: 60 ng/mL). This concentration fully blocked Wnt activity in functional Wnt reporter assays on NSCs (Walter et al. 2024).

The plate was returned to the xCELLigence station, and CIs values were measured every 5 min for 30 min. The wells were then treated with 10 nM MPH. The untreated well’s vehicle was water. The same method as the previous experiments was used to calculate proliferation rates under MPH treatment.

### Wnt reporter assay in NSCs after acute MPH treatment

Wnt reporter assays were conducted in both ADHD and control NSCs, following the transfection-based methodology outlined in Yde Ohki et al. (2023b). In this protocol, NSCs were co-transfected with the plasmid of interest containing a Wnt luciferase reporter gene under the control of a TCF/LEFresponsive element (*pGL4*.*49*[luc2P/TCF-LEF RE/Hygro] Vector, from Promega) and a normalization vector containing the NanoLuc luciferase gene under the control of a TK (thymidine kinase) promoter (pNL1.1.TK[Nluc/TK] Vector, Promega).

After previous overnight treatment with the Wnt-agonist Wnt3a, EC_50_ values were determined for each cell line in two technical replicates (Yde Ohki et al. 2023b). According to the previous results (Walter et al. 2024), NSCs were treated individually with the respective Wnt3a’s EC_25_ in triplicates. Subsequently, they were returned to a cell culture incubator set at a temperature of 37 °C and 5% CO_2._

After 10 min, 10 nM MPH was added to the wells. The condition named “Vehicle” represents treatment with individual EC_25_ concentrations of Wnt3a, only. In this context, water was used as vehicle for MPH treatment.

Next, the cells were incubated overnight at 37 °C and 5% CO_2._ On the following day, luminescence assays and subsequent data analysis from Relative Luminescence Units (RLU) were performed as previously reported in Yde Ohki et al. (2023b). Experiments with MPH were also conducted in two technical replicates for each cell line.

### Western blot of Wnt-related proteins following chronic MPH treatment

Since performing Wnt reporter assays after chronic MPH treatment would not represent a comparable and consistent method with other functional analyses in the Wnt pathway conducted by our group (Walter et al. 2024, 2025), Western Blot analyses were conducted after administering MPH for seven consecutive days to investigate the potential effects of chronic MPH on the expression of key Wnt-proteins, including LRP6, active β-catenin, and total and phosphorylated GSK3β, which may be associated with proliferation outcomes.

When one well from a 6-well plate (Sarstedt) containing NSCs reached 100% confluence, they were harvested and subsequently seeded at a 1:3 to 1:6 dilution ratio into new 6 wells coated with Geltrex (Gibco™) diluted in DMEM/F12 (Gibco™) in a ratio of 1:100 and incubated for 1 h at 37 °C. Throughout this 7-day period, cells were cultured at 37 °C and 5% CO_2_, and were treated daily with water as vehicle or MPH at 10 nM or 100 nM. Specifically, the wells were completely refreshed with fresh media, which consisted of NEM with MPH on days 1, 3, 5 and 7 post-seeding, while MPH was only added to the wells on days 2, 4 and 6 post-seeding. On day 8 post-seeding, NSCs were harvested using StemPro^®^ Accutase^®^ (Gibco™), and after centrifugation at 300 × g for 4 min, proteins were extracted using 1% Halt™ Protease & Phosphatase Single-Use Inhibitor Cocktail (Thermo Fisher Scientific™) in RIPA Buffer (Thermo Fisher Scientific™).

Colorimetric detection quantitation of total protein concentration was measured for each cell line in triplicate according to the Microplate procedure using the Pierce™ BCA Protein Assay Kit (Thermo Fisher Scientific™).

Every protein sample (5 μg) was incubated in Bolt™ LDS Sample Buffer 1X (Thermo Fisher Scientific™) and Bolt™ Sample Reducing Agent 1X (Thermo Fisher Scientific™) for 10 min at 70 °C. Bolt™ 4–12% Bis–Tris Plus Gels and the XCell SureLock Mini-Cell Electrophoresis System (Thermo Fisher Scientific™) were used, and the gel was run at constant 200 V for 40 min. The transfer of gels onto iBlot^®^ nitrocellulose membranes (Thermo Fisher Scientific™) was performed for 7 min at 20 V using the iBlot^®^ Gel Transfer Device (Thermo Fisher Scientific™).

The Pierce™ Fast Western Blot Kit, ECL Substrate (Thermo Fisher Scientific™) was used to stain the membrane for proteins involved in the Wnt/β-catenin pathway such as LRP6, active β-catenin, and total and phosphorylated GSK3β. Details about the primary and secondary antibodies used in this protocol may be found in Supplementary Table 3. Membranes were incubated with primary antibodies diluted in antibody buffer for 20 h at 4 °C. On the next day, an incubation with secondary antibodies diluted 1:10,000 in antibody buffer was performed. The membrane was imaged by the Molecular Imager^®^ ChemiDoc™ XRS + using the Chemi Hi Resolution set for 30 s, 45 s, 60 s and 75 s and the protein ladder (PageRuler Prestained Protein Ladder; 10–180 kDa) was detected using the colorimetric application. All images were analyzed in ImageLab 6.0, and glyceraldehyde-3-phosphate dehydrogenase (GAPDH) was used as a housekeeping protein. As an additional normalization factor, a protein sample from the cell line MR010 c3 was used as an internal control. For each condition, two protein samples per cell line were analyzed in two independent experiments.

### Data and statistical analysis

According to the iPSC-based tool developed by Brunner et al. (2023), assuming an effect size (Cohen’s *d*) of 1.0 [based on a study using iPSCs with similar readouts and research questions (Papes et al. 2022)] and a moderate intercluster correlation of 0.15, 8 or 10 cell lines would provide sufficient statistical power of 0.8 for the main outcome of the investigation of MPH effects in NSCs (which includes at least 52 observations per cell line). Thus, N = 10 cell lines per group was chosen to ensure a statistical power above 0.8. Each group included 5 individuals (2 iPSC clones) analyzed in all experiments. Western Blot band intensities were analyzed with ImageLab 6.0, while absorbance and luminescence data were measured with MikroWin 2010 (version 5.18). R (version 4.4.1) and RStudio (version 2023.6.1.524) were used to perform all statistical tests. The graphs (except for the correlation matrix) were generated with GraphPad Prism (San Diego, CA, USA; version 10.3.0). 

Supplementary Table 4 contains the list of experiments to which our NSC lines were submitted, as well as appropriate references from previous publications, when applicable.

We used the *lmer* function from the ‘lme4’ package in R to perform linear mixed-effects modeling with fixed effects and a nested random intercept (1| Cell/Replicate) to account for nested data structure. Satterthwaite-approximated degrees of freedom were used to generate p-values. Simulation in the ‘DHARMa’ package was used to test model assumptions. When assumptions were violated, robust models (the *rlmer* function from the ‘robustlmm’ package) were used. *Post hoc* comparisons with Tukey’s correction were applied to explore pairwise differences between fixed effect levels if a main effect or interaction was statistically significant (or tending toward significance) using the ‘emmeans’ package. Supplementary Table 5 shows *post hoc* test results. Since only one clone per donor was considered in ADHD and control groups (Supplementary Fig. 3), Mann–Whitney tests were applied to compare baseline *versus* water conditions. 

The bar graphs represent each clone separately because cell culture introduces genetic and epigenetic differences between clones derived from the same subject and genetic background (Sgodda and Cantz 2013; Liang and Zhang 2013).

The graphs depict model-adjusted means ± standard error of the mean (SEM). All bar charts show the average of two independent replicates per cell line as dots. Asterisks indicate statistical significance (*p* < 0.05), while hashtags indicate a trend toward statistical significance (p-values between 0.05 and 0.075).

As previously described (Walter et al. 2024), Spearman’s correlations were computed as a matrix of clinical, genetic, and cellular variables. Both uncorrected and Bonferroni-corrected matrices were presented.

## Results

### ADHD NSCs proliferate at a slower rate than controls

**Fig. 1:**
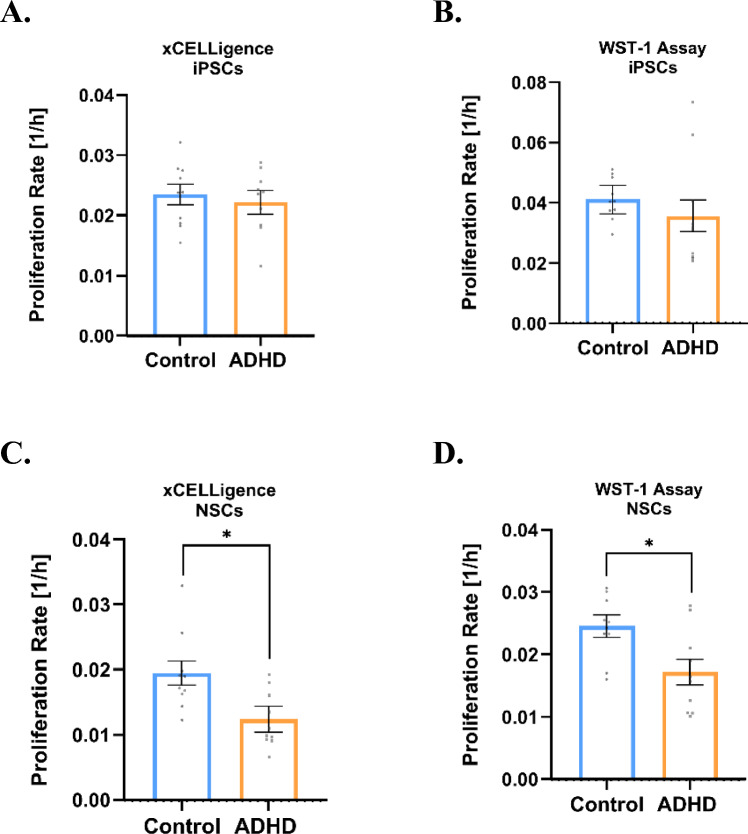
Proliferation analysis in ADHD and control cell lines. In both xCELLigence (**A**) and WST-1 assays (**B**), no statistically significant (n.s.) differences were observed between ADHD and control iPSCs. For xCELLigence: standard *lmer*, estimate (Est.) = 0.001, standard error (SE) = 0.003, *t*(15.81) = 0.50, *p* = 0.627; for WST-1: standard *lmer*, Est. = 0.005, SE = 0.007, *t*(4.26) = 0.81, *p* = 0.463. N = at least 2 technical replicates were analyzed for each cell line (5 individuals per group, 2 clones each). In NSCs, lower proliferation rates in ADHD are seen for both xCELLigence (**C**) and WST-1 (**D**). For xCELLigence: standard *lmer,* Est. = − 0.007, SE = 0.003, *t*(8.00) = − 2.74, *p = 0.025. For WST-1: standard *lmer,* Est. = − 0.007, SE = 0.003, *t*(16.15) = − 2.72, *p = 0.015. In xCELLigence, the average of 4 vehicle experiments was calculated per cell line. N = 5 control (2 clones each) and 5 ADHD patients (2 clones each) were analyzed in 2 technical replicates. Each dot represents the averaged raw data obtained from independent experiments per cell line. Mean ± SEM (model-adjusted, standard *lmer*) is depicted in the graphs

Both of our proliferation methods (xCELLigence and WST-1 assays) showed that proliferation rates of iPSCs did not significantly differ between ADHD and control groups (Fig. 1A, B). Individual proliferation rates for iPSCs in xCELLigence and WST-1 assays are provided in Supplementary Figs. 2A and 2B, respectively.

However, at the neurodevelopmental stage of NSCs, the ADHD lines exhibited a significantly lower rate of proliferation than controls in both the xCELLigence (Fig. 1C) and WST-1 methods (Fig. 1D).

We previously observed that proliferation of vehicle-treated NSCs (water) did not statistically differ from the baseline results (completely untreated) when analyzing 4 ADHD lines and 4 control lines (Supplementary Fig. 3). Consequently, the findings depicted in Fig. 1C, 1D, 5, and Supplementary Figs. 4A, 5A, and 7 pertain to water-treated NSCs.

### MPH treatment at a low concentration modestly modulates proliferation of ADHD NSCs

**Fig. 2:**
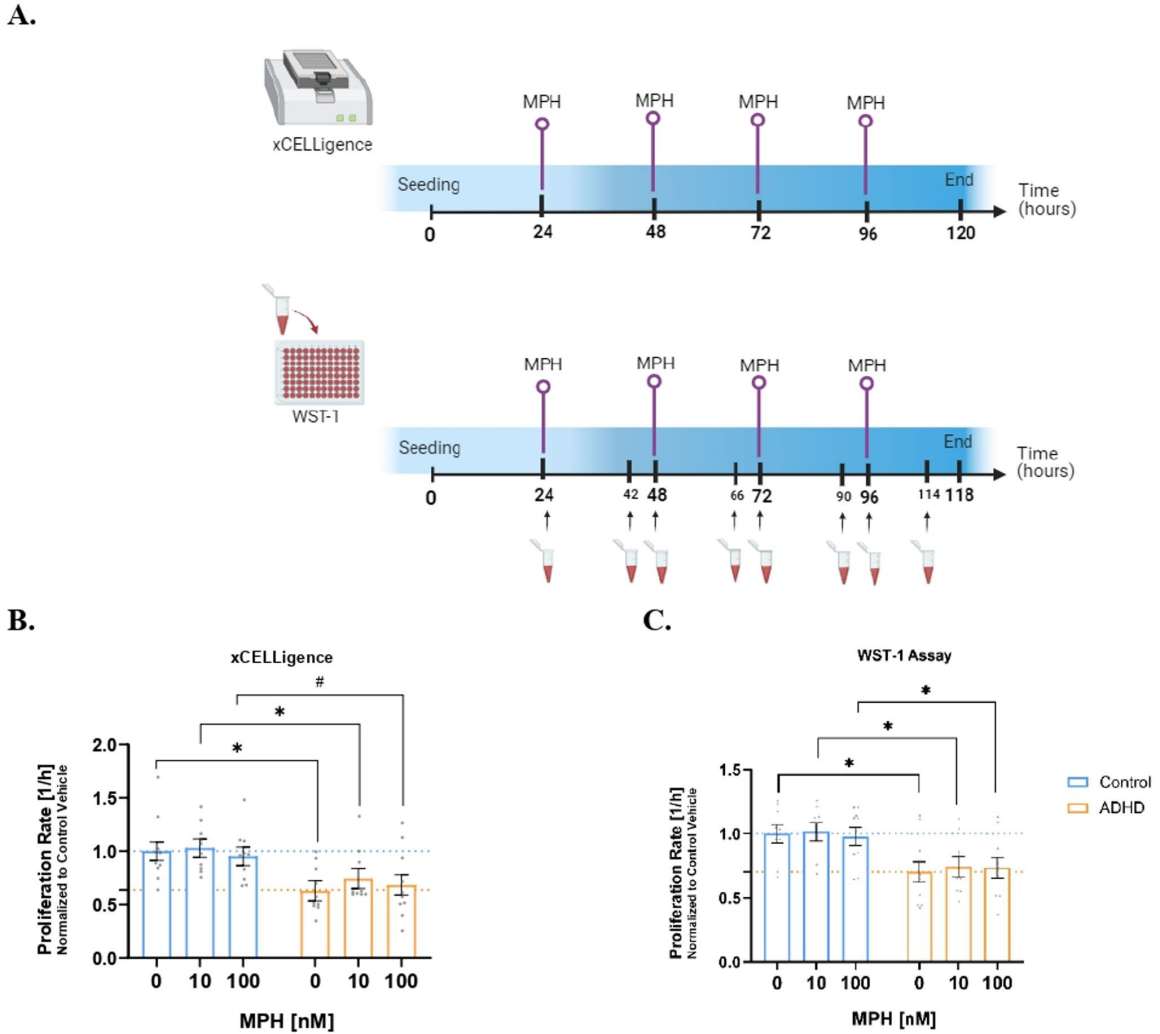
Differences in proliferation rates between ADHD and control NSCs with and without MPH treatment. **A** Experimental design of xCELLigence and WST-1 with daily MPH treatments. The red tubes represent the addition of WST-1 at each timepoint (figure created with Biorender.com). Absorbance measurements took place 4 h later. The bar graphs represent proliferation rates obtained by xCELLigence (**B**) or WST-1 assays (**C**) after 4-day MPH treatment. Statistically significant *post hoc* comparisons are depicted by asterisks, whereas hashtags represent trends toward significance (for xCELLigence: standard *lmer*, *p = 0.012 for ADHD *versus* Control at Vehicle, *p = 0.043 for ADHD *versus* Control at MPH 10 nM and ^#^*p* = 0.055 for ADHD *versus* Control at MPH 100 nM; for WST-1: standard *lmer*, *p = 0.020 for ADHD *versus* Control at Vehicle, *p = 0.029 for ADHD *versus* Control at MPH 10 nM and *p = 0.046 for ADHD *versus* Control at MPH 100 nM). Four vehicle experiments were considered and averaged per cell line. Mean ± SEM (model-adjusted, standard *lmer*) is depicted in the graphs. N = 5 Control individuals (2 clones each) and 5 ADHD patients (2 clones each) were analyzed in 2 technical replicates. Each dot represents the averaged raw data from 2 independent experiments per cell line

In the next step, NSCs were treated with MPH on a daily basis to examine any potential rescue effects of this drug on their proliferation (Fig. 2A). A significant effect of diagnosis was found in both xCELLigence (standard *lmer,* Est. = 0.37, SE = 0.13, *t*(15.49) = 2.93, *p = 0.010) and WST-1 (standard *lmer,* Est. = 0.30, SE = 0.11, *t*(19.74) = 2.84, *p = 0.010). Diagnosis-wise *post hoc* tests were applied to data generated from both methods to verify differences between ADHD and control groups at each MPH dose. After treatment in xCELLigence, the notable differences observed at basal level continued and MPH did not raise the proliferation rates of the ADHD group to the control levels, which was evidenced by the pairwise comparisons between groups (Fig. 2B; Supplementary Table 5).

However, in terms of percentage change, there were indications of increased rates of proliferation, specifically when ADHD NSCs were treated with a concentration of 10 nM in xCELLigence. When applied to ADHD cell lines, this led to an approximate 18% (statistically non-significant) increase in proliferation rates beyond their baseline rates, whereas the dose of 100 nM resulted in a lower change in proliferation in this group corresponding to ca. 8.6% (Fig. 2B). The proliferation of the control group increased by only 3% after treatment with MPH at 10 nM compared to its vehicle (Fig. 2B). In contrast, 100 nM MPH slightly decreased proliferation by 4.8% in the control group compared to its vehicle (Fig. 2B).

Similarly, statistically significant differences between groups were observed for each MPH concentration in WST-1 (Fig. 2C; Supplementary Table 5). In terms of percentage change, MPH-treated ADHD NSCs at 10 nM showed a 5.7% increase compared to ADHD vehicle, although this was statistically non-significant. MPH at 100 nM increased proliferation of ADHD NSCs by only 4.7% compared to its vehicle, while moderately reducing proliferation rates in control NSCs relative to control vehicle, resulting in a statistically non-significant decrease of 2.2% (Fig. 2C). Supplementary Figs. 4A-C and 5A-C show individual proliferation rates before and after MPH treatment.

### Blocking the Wnt pathway with DKK1 alters the proliferation-related effects of MPH treatment in ADHD NSCs

**Fig. 3:**
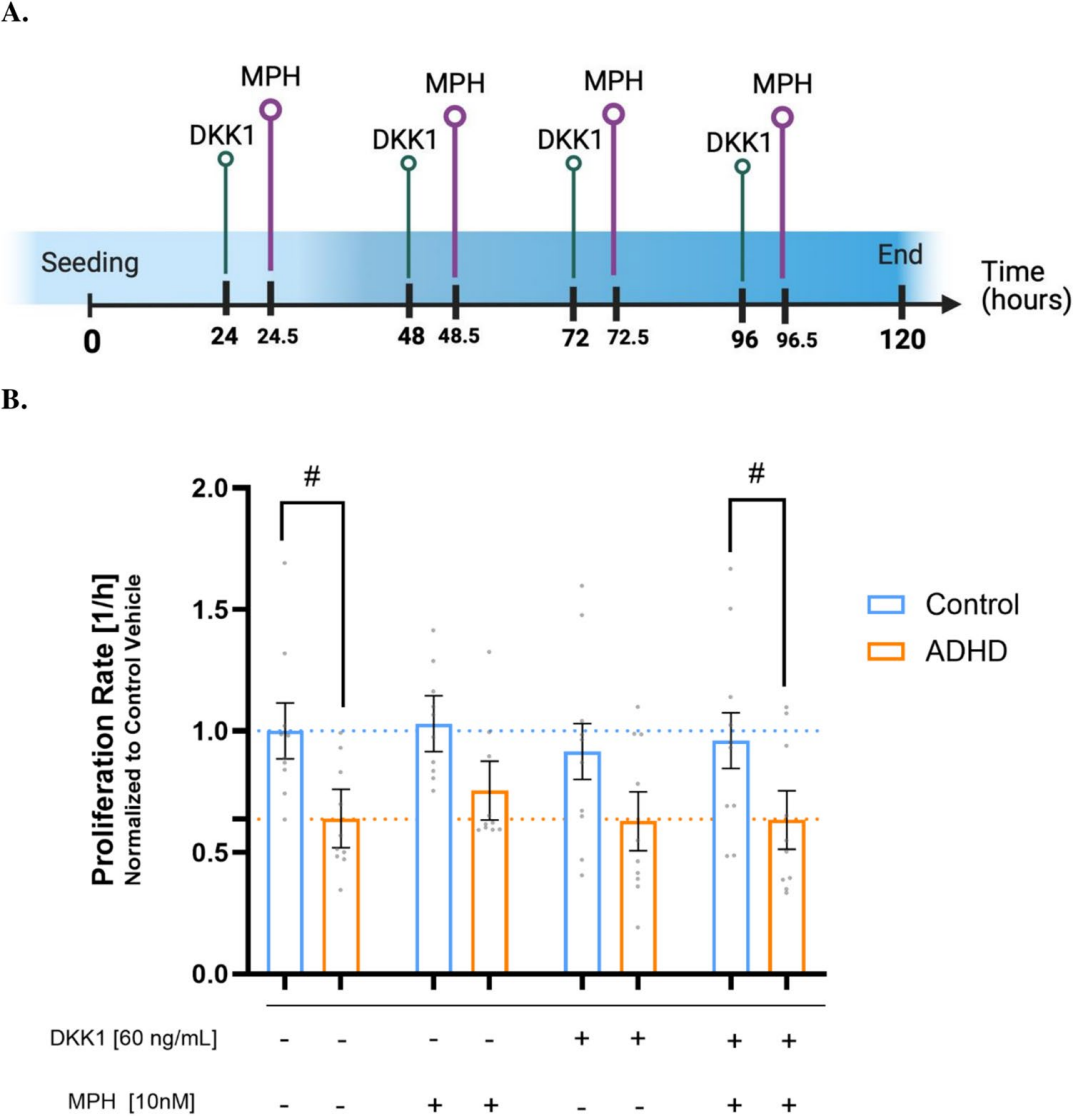
NSC proliferation after blockade of Wnt activity using DKK1 60 ng/mL. **A** Timeline representing the design of xCELLigence experiments, in which proliferation of NSCs was tested throughout 5 days following a four-day treatment with DKK1 and/or MPH. Water was used as the vehicle for MPH when only DKK1 was added to the wells. Figure created with BioRender.com. **B** Proliferation rates of NSCs after daily treatment with DKK1 and/or MPH. Hashtags represent trends toward differential responses between ADHD and control groups (standard *lmer,* ^#^*p* = 0.051 for Vehicle and ^#^*p* = 0.073 for MPH 100 nM). For vehicle conditions in xCELLigence, the average of 4 vehicle experiments was calculated per cell line. All other conditions were normalized to Control Vehicle. Mean ± SEM (model-adjusted, standard *lmer*) is depicted in the graph. N = 5 Control individuals (2 clones each) and 5 ADHD patients (2 clones each) were analyzed in 2 technical replicates. Each dot represents the averaged raw data from 2 independent experiments per cell line

Given previous studies demonstrating the ability of MPH to regulate the canonical Wnt pathway (Grünblatt et al. 2018; Yde Ohki et al. 2020), we postulated that DKK1 could be effective in reducing the increase in ADHD NSC proliferation induced by a concentration of 10 nM MPH. To determine whether there are any associations between MPH, Wnt, and proliferation, proliferation rates were measured using xCELLigence for five days after MPH treatment. However, 30min prior to MPH treatment, DKK1 at 60 ng/mL was used to inhibit Wnt signaling. Figure 3A illustrates the experimental design for four treatment cycles of DKK1 and/or MPH.

The effect of diagnosis was found to be significant (standard *lmer,* Est. = 0.36, SE = 0.17, *t*(13.52) = 2.17, *p = 0.049). Diagnosis-wise *post hoc* tests revealed trends toward significance between Control and ADHD at the basal level (^#^*p* = 0.051) and after combined treatment with DKK1 and MPH (^#^*p* = 0.073). However, no significant differences were observed between groups when NSCs were treated with MPH alone (*p* = 0.124) or DKK1 alone (*p* = 0.110) (Fig. 3B; Supplementary Table 5).

While MPH increased ADHD proliferation rates, DKK1 treatment did not enhance these rates and instead reduced them in the control group. Notably, the proliferative effects of MPH on ADHD NSCs were abolished when the Wnt signaling pathway was blocked, compared to the proliferation rates observed when the NSCs were treated with MPH alone (Fig. 3B; Supplementary Table 5).

### Functional findings indicate upregulated Wnt activity by acute MPH 10 nM in ADHD NSCs

**Fig. 4:**
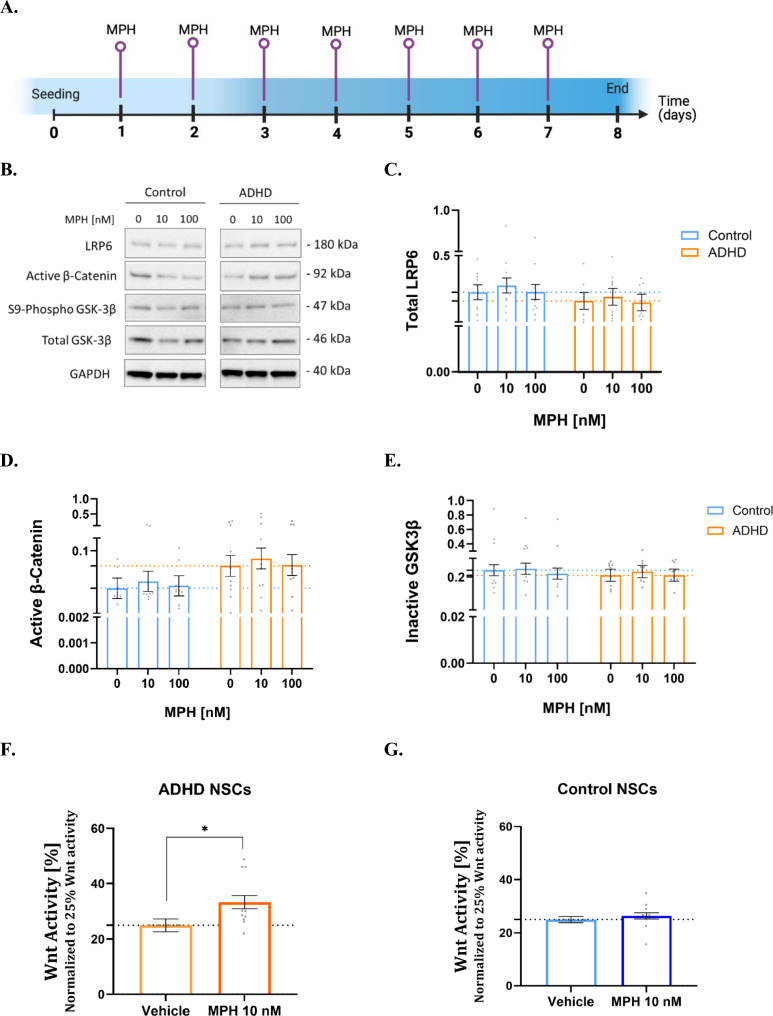
Analysis of MPH influence on Wnt signaling of NSCs from ADHD patients and controls. **A** Timeline of Western Blot experiments. Figure created with BioRender.com. **B** Representative bands from one control NSC line (K011 c10) and one ADHD NSC (MR010 c18) for all analyzed Wnt-related proteins after chronic treatment with 10 nM and 100 nM MPH. GAPDH is shown as reference. Total levels of LRP6 (**C**), active β-catenin (**D**), and inactive GSK3β (**E**) were quantified before and after 7-day MPH treatment. No significant effects or trends were observed. Group-specific Wnt activity in ADHD (**F**) and control NSCs (**G**) was measured using the reporter assay after acute treatment with 10 nM MPH. For ADHD NSCs: standard *lmer*, Est. = -8.36, SE = 3.06, *t*(18.00) = -2.73, *p = 0.014; for control NSCs: standard *lmer*, Est. = -1.41, SE = 1.55, *t*(14.00) = − 0.91, *p* = 0.378. In **F** and **G**, bar charts show levels of Wnt activity after treatment with MPH, normalized to their respective vehicles representing 25% of Wnt activity. Mean ± SEM (model-adjusted, standard *lmer* for LRP6 and Wnt reporter findings; robust *lmer* for active β-catenin and inactive GSK3β) is depicted in the graph. N = 5 control individuals (2 clones each) and 5 ADHD patients (2 clones each) were analyzed in 2 technical replicates. Each dot represents the averaged raw data from 2 independent experiments per cell line

Given that patients usually take MPH daily, we aimed to investigate whether chronic MPH treatment was able to induce changes in the expression of specific proteins that compose the Wnt/β–catenin pathway. To this end, we performed Western Blot experiments after 7 daily MPH treatments at 10 nM and 100 nM (Fig. 4A, 4B). Expression of total LRP6 (Fig. 4C), active β–catenin (Fig. 4D), and inactive GSK3β (Fig. 4E) was measured for all conditions. Inactive GSK3β levels were calculated as the ratio of S9-phosphorylated to total GSK3β. For all three proteins, no statistically significant effects or trends were observed for any variable in this study (diagnosis, MPH concentrations, or their interaction), based on the standard *lmer* model involving expression of total LRP6 and the robust *lmer* model involving active β–catenin (Fig. 4D) and inactive GSK3β (Fig. 4E).

However, we noticed a non-significant increase in active β-catenin levels (an 85% increase *versus* controls) at the basal state (vehicle) in ADHD compared to controls. Consequently, we compared the basal levels of this protein between ADHD and control NSCs. As opposed to LRP6 and inactive GSK3β (Supplementary Figs. 6A and 6C), we observed a tendency toward increased levels of active β-catenin in ADHD NSCs, although this difference was not statistically significant (Supplementary Fig. 6B; standard *lmer*, ^#^*p* = 0.068).

Using functional Wnt reporter assays (Yde Ohki et al. 2023b), Wnt activity in ADHD NSCs was assessed after acute treatment with MPH at 10 nM. To assess the impact of MPH at a concentration of 10 nM compared to the baseline state, both the control and ADHD groups were subjected to a baseline level of 25% Wnt activity. Therefore, their vehicle conditions are not being compared. Previously, we have shown that ADHD cell lines had overactive Wnt activity in comparison to controls, which was indicated by Western Blot and Wnt reporter results (Walter et al. 2024). Thus, the goal of this experiment was to solely identify any modulatory effects of MPH. In our findings, ADHD NSCs demonstrated a significant increase in Wnt activity following MPH treatment at 10 nM compared to the vehicle (Fig. 4F). Conversely, 10 nM MPH   did not enhance Wnt activity in the control group (Fig. 4G).

### Multiple associations between genetic, cellular and clinical parameters were found

**Fig. 5:**
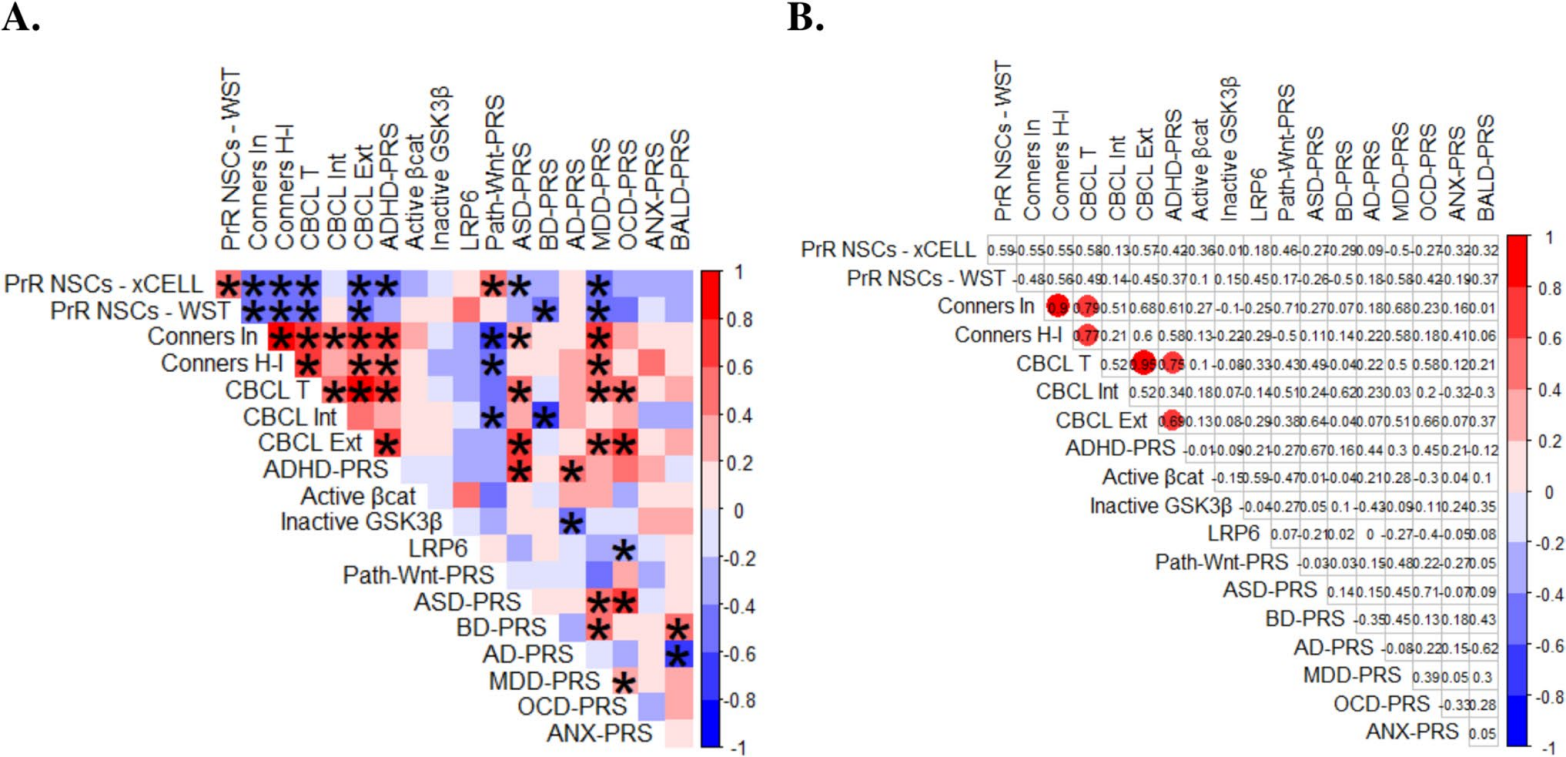
Correlations between ADHD-related behavioral scores, Wnt-PRS, PRS for ADHD and other disorders, and *in vitro* findings. **A** Uncorrected pairwise Spearman’s correlations are shown in the plot. Colors code the strength and direction of each correlation, whereas asterisks indicate statistically significant correlations without correction (*p* < 0.05). **B** After the Bonferroni correction for 171 unique pair combinations (0.05/171), 6 pairs survived the correction (*p* < 0.05): Conners In *versus* Conners H-I, Conners In *versus* CBCL T, Conners H-I *versus* CBCL T, CBCL T *versus* CBCL Ext, CBCL T *versus* ADHD-PRS, CBCL Ext *versus* ADHD-PRS. The red color in these comparisons indicates positive correlations while the numbers represent their respective correlation coefficients. Abbreviations: PrR NSCs– xCELL: Proliferation rates of NSCs from xCELLigence assays; PrR NSCs– WST: Proliferation rates of NSCs from WST-1 assays; Conners In = Inattention scores from Conners’ Rating Scales; Conners H-I: Hyperactivity/Impulsivity scores from Conners’ Rating Scales; CBCL T = Total Scores from CBCL; CBCL Int = Internalizing scores from CBCL; CBCL Ext = Externalizing scores from CBCL; Active βcat = Protein expression of active β-catenin after a 7-day cell culture; Inactive GSK3β = Protein expression of inactive GSK3β after a 7-day cell culture; LRP6 = Protein expression of LRP6 after a 7-day cell culture; ADHD-PRS = Polygenic Risk Scores for Attention-Deficit Hyperactivity Disorder; Path-Wnt-PRS = Pathway-PRS specific to Wnt signaling; ASD-PRS = Polygenic Risk Scores for Autism Spectrum Disorder; BD-PRS = Polygenic Risk Scores for Bipolar Disorder; AD-PRS = Polygenic Risk Scores for Alzheimer’s Disease; MDD-PRS = Polygenic Risk Scores for Major Depressive Disorder; OCD-PRS = Polygenic Risk Scores for Obsessive–Compulsive Disorder; ANX-PRS = Polygenic Risk Scores for Anxiety Disorder. Genetic variables were also present in a previous publication (Walter et al. 2024)

As anticipated, due to the same pattern of results provided by xCELLigence and WST-1 assays, proliferation rates derived from these methods correlated significantly and positively with one another (Fig. 5A). Slower proliferation was shown to be significantly associated with higher clinical Conners' Rating Scales for hyperactivity, impulsivity, and inattention, as well as with externalizing or total scores from CBCL (Fig. 5A).

Consistent with previous large population studies (Green et al. 2022), there was a significant positive correlation between ADHD-PRS and CBCL’s externalizing and total scores, as well as between ADHD-PRS and Conners’ scores for hyperactivity and impulsivity. Furthermore, there was a negative correlation between NSC proliferation rates (measured by both xCELLigence and WST-1) and individual ADHD-PRS (*p* = 0.025 and *p* = 0.097, respectively) (Fig. 5A, Supplementary Table 6). Overall, we identified negative associations between proliferation rates and genetic susceptibility to other neuropsychiatric illnesses, such as ASD, BD, and MDD.

Genetic liability to ADHD also correlated positively to ASD- and AD-PRS in a statistically significant manner, while no correlation was observed for ADHD *versus* MDD, OCD, ANX, or BD (Fig. 5A). The Wnt-specific PRS exhibited a substantial negative correlation with ADHD-related behavioral scores, such as Conners’ hyperactivity/impulsivity and inattention and CBCL inattention, as shown in Fig. 5A. The correlation between Wnt-PRS and ADHD-PRS was negative, with a nominal significance of *p* = 0.058 (Supplementary Table 6). In contrast, baldness-related PRS expectedly did not correlate with ADHD symptomatology or any cellular variables, as also previously described (Walter et al. 2024) (Fig. 5A).

Upon thorough examination of the interconnections among the three analyzed Wnt elements, it is evident that a positive correlation exists, albeit not statistically significant, between the expression of active β-catenin and total LRP6. This implies the potential existence of a positive feedback loop mechanism (Fig. 5A).

While our correlation analyses are exploratory in nature, the large number of comparisons (171) increases the risk of Type I errors. Therefore, we present the correlation matrices both uncorrected (Fig. 5A) and after Bonferroni correction (Fig. 5B). Supplementary Table 6 reports both uncorrected and corrected p-values. Only six pairs survived the correction: (1) Conners Inattention (Conners In) scores *versus* Hyperactivity/Impulsivity scores (Conners H-I); (2) Conners In *versus* rstotal problems scores from the CBCL (CBCL T); (3) Conners H-I *versus* CBCL T; (4) CBCL T *versus* externalizing problems scores from the CBCL (CBCL Ext); (5) CBCL T *versus* ADHD-PRS; and (6) CBCL Ext *versus* ADHD-PRS (Fig. 5B).

To further explore the results while eliminating the issue of pseudoreplication due to the representation of two clones from one individual in the matrix, we averaged the *in vitro* results from both clones per individual and recalculated the correlations with N = 10 (Supplementary Fig. 7, Supplementary Table 7). Although ADHD-PRS no longer correlated with AD-PRS in a statistically significant manner (*p* = 0.120) (Supplementary Table 7), we observed that similar patterns of correlations were preserved when compared to Fig. 5A. These include: (1) proliferation rates obtained from xCELLigence and WST-1 assays, (2) different clinical scores of ADHD symptomatology overall, (3) clinical scores *versus* ADHD-PRS, (4) ADHD-PRS *versus* ASD-PRS, (5) Wnt-PRS *versus* Conners’ scores for inattention, (6) PRS of distinct neuropsychiatric disorders *versus* ADHD-related cellular findings and clinical scores, and (7) NSC proliferation rates *versus* clinical scores (Supplementary Fig. 7A, Supplementary Table 7). However, only the correlation between CBCL scores for inattention and CBCL total scores survived the conservative Bonferroni correction in this analysis (Supplementary Fig. 7B, Supplementary Table 7).

All in all, despite the exploratory nature of these analyses, the results may inspire novel hypotheses to be tested in the future.

## Discussion

This study expands on our previous research on iPSC-derived cell line proliferation rates in ADHD patients and controls by employing a larger sample size and including female participants. It demonstrates variation in NSC cell proliferation, rather than in iPSCs (Yde Ohki et al. 2023a). These results support the notion that ADHD is a brain development disorder and that gene expression profiles can change temporally and spatially (Smith et al. 2010; Weyn-Vanhentenryck et al. 2018), resulting in cell type-specific phenotypes.

The etiology of ADHD is often linked to reduced cortical thickness (Narr et al. 2009). This study suggests that clinical brain maturational delays in ADHD may be caused by impaired NSC cell proliferation. Thyroid hormone-responsive protein-overexpressing (THRSP-OE) mice, an ADHD animal model, show lower hippocampal cell proliferation (Custodio et al. 2023). IPSC-derived brain organoids from one male ADHD patient exhibited reduced thickness in the cortical plate and ventricular zone (Zhang et al. 2023). Despite distinct views on postnatal NSC proliferation and neurogenesis, proliferative adult NSCs may still populate the mammalian brain’s subventricular zone, undergo neurogenesis/astrogenesis, and transiently persist in the cortex after injury (Faiz et al. 2015). Ohira thoroughly discussed the presence of cortical NSCs and neural progenitor cells (NPCs) in 2018 (Ohira 2018). Based on this, we hypothesize that postnatal proliferation of NSCs may be affected in ADHD in a non-embryonic stage and modulated by MPH.

We found that the genetic susceptibility to ADHD should be considered when studying NSC proliferation, consistent with our Wnt-related protein expression findings (Walter et al. 2024). Albeit exploratory, our data indicate a negative correlation between NSC proliferation and ADHD-PRS. A high polygenic load for this disorder has been associated with reduced intracranial variance and cortical surface area (Liu et al. 2023). Our previous study (Walter et al. 2024) found a positive correlation between ADHD-PRS and ADHD-related behavioral traits, and now we show a negative correlation between those clinical features and NSC proliferation. Multiple clinical studies have linked genetic susceptibility to ADHD to clinical traits like impulsivity (Barker et al. 2021, Martin et al. 2014; Sudre et al. 2020). Cortical thickness in cognitive functioning regions, such as in the right anterior attention network, may predict ADHD symptoms (Bledsoe et al. 2013). Castellanos et al. found a substantial correlation between ADHD severity and lower cerebral volumes (e.g., from cerebellum, frontal and temporal gray matter, and caudate) (Castellanos et al. 2002).

The same correlation was observed when PRS for ASD and MDD were compared to NSC proliferation rates. Due to genetic overlap and high comorbidity, these disorders are correlated (Lee et al. 2019). Consistently, as expected, individuals who are genetically more prone to develop ADHD showed higher clinical and behavioral scores, which was also observed for ASD- and MDD-PRS in a statistically significant manner. We observed a trend toward a positive correlation between ANX predisposition and hyperactivity/impulsivity. However, contrary to our expectations, associations between ANX-PRS and other parameters were not as insightful, given the high prevalence of comorbidities between ANX and ADHD (Koyuncu et al. 2022).

As previously discussed (Walter et al. 2024), the strong genetic association between AD, a neurodegenerative disorder, and ADHD is one of the strongest pieces of evidence that these two conditions are strongly related in terms of cellular and clinical outcomes (Leffa et al. 2023; Grünblatt et al. 2023). Numerous studies have linked Wnt downregulation to cognitive dysfunctions in AD (Palomer et al. 2019; Jia et al. 2019), supporting the negative correlation between inactive GSK3β levels and AD-PRS observed in this study.

Increased Wnt-PRS tended to correlate with NSC proliferation. This finding corroborates our hypothesis that Wnt-related genetic variations regulate its function, protect, and promote neurodevelopment (Walter et al. 2024). This is also consistent with the observed trend of negative correlations between Wnt-PRS and ADHD-PRS in this study, which may indicate a close relationship between this pathway and ADHD, as hypothesized (Yde Ohki et al. 2020). Conversely, the lack of significant correlations between ADHD-related clinical scores, cellular variables, and genetic predisposition to baldness, a non-psychiatric disorder, strengthens the specificity of our findings.

Only six correlation pairs between PRS and ADHD-related CBCL and Conners clinical scores survived Bonferroni correction. Among these, when clone data were averaged, only the correlation between CBCL T and CBCL Ext remained significant. Altogether, this shows a strong association between ADHD symptoms and genetic predisposition. This supports previous studies showing that ADHD-PRS correlates with symptom severity (Agnew-Blais et al. 2021; Saraçaydın et al. 2024) and confirms that our ADHD patients and controls were recruited consistently.

A negative correlation between active β-catenin levels and NSC proliferation results obtained using xCELLigence showed a trend toward significance (Supplementary Table 6). Stabilized β-catenin leads to NPC expansion *in vivo* (Chenn and Walsh 2002), but the Wnt/β-catenin pathway is also responsible for maintaining NSC homeostasis, including cell fate specification and differentiation (Gao et al. 2021).

Our data also showed trending correlations between CBCL internalizing scores and xCELLigence or WST-1 results. Technical inconsistencies between the two approaches may cause differences in results, as elaborated in our preliminary publication (Yde Ohki et al. 2023a). Both methods yielded similar results, but WST-1 tests indirectly measure proliferation at a single timepoint, while xCELLigence measures CIs from individual wells over time (Yde Ohki et al. 2023a). This may render xCELLigence measurements more representative of NSC proliferation.

As hypothesized, MPH did not completely correct the differences in NSC proliferation between ADHD and control groups. The drug's four-day administration, compared to patients’ months or years, may explain its limited ability to stimulate ADHD NSC cell proliferation. Nevertheless, we found that the ADHD group showed proliferative improvement, as seen in clinical observations (Nakao et al. 2011).

Our group previously found that MPH promoted neuronal differentiation at the expense of cell proliferation in murine NSCs (Bartl et al. 2013). This study contradicts that finding. However, species-specific cell profiles must be considered. Further research demonstrated that MPH activated Wnt signaling to decrease proliferation and increased differentiation of rat PC12 and human SH-SY5Y neuroblastoma cells (Grünblatt et al. 2018). The Wnt pathway is thought to be conserved across species (Croce and McClay 2008), but differences in Wnt signaling between human and rat cells may explain past and present findings. These differences may include Wnt-related gene expression (Gonzalez-Fernandez et al. 2017) and Wnt dynamics across cell types (Sethi and Vidal-Puig 2010).

Our recent study found that ADHD NSCs have higher Wnt activity than controls (Walter et al. 2024). This was confirmed by Wnt reporter assays (Walter et al. 2024) and expression analysis of the same proteins analyzed in our current study. A tendency toward increased levels of active β-catenin were observed in ADHD NSCs after continuous administration of 10 nM MPH, although the increase was not statistically significant. Given the absence of statistical significance, these data should be interpreted with caution and the implicated mechanisms should be further clarified. These preliminary results may be a starting point for further research into whether chronic MPH treatment at lower doses enhances Wnt activity. *In vivo* studies have shown that a 28-day MPH treatment at lower doses (1 mg/kg) increases β-catenin levels and hippocampal cell proliferation in mice (Oakes et al. 2019). This evidence supports the current findings. A tenfold higher dose reduced protein expression during the same time and favored neuronal differentiation (Oakes et al. 2019).

Functional experiments revealed that only ADHD NSCs exhibited elevated Wnt activity after acute MPH (10 nM) treatment. The study found a non-significant increase in active β-catenin levels in ADHD cells after administering 10 nM MPH, and a slight decrease at 100 nM over 7 days. MPH-related benefits may persist over long-term treatment, but more research is needed. After 2 h, 10 nM falls within the physiological range observed in humans, which is the pharmacodynamic point when MPH peaks in blood plasma (Wargin et al. 1983; Preiskorn et al. 2018). MPH 10 nM treatment may temporarily increase ADHD NSC proliferation by increasing Wnt activity, as β-catenin has been shown to stimulate cell division of human NSCs (Gao et al. 2021). This evidence suggests this discovery may be clinically significant.

DKK1-induced Wnt inhibition prior to MPH treatment in ADHD NSCs eliminated MPH-induced effects in these cell lines, indicating that they are dependent on the Wnt signaling. Due to extensive intracellular interactions between Wnt and other proliferation-regulating pathways like Notch, fibroblast growth factors (FGF), and brain-derived neurotrophic factors (BDNF) (Lee et al. 2021b; Yang et al. 2016; Tang et al. 2019), more research is needed to determine how MPH affects the Wnt cascade.

Distinct hypotheses can be drawn regarding the mechanisms by which MPH targets the Wnt pathway. Previous studies have indicated that MPH may also affect adjacent signaling pathways related to Wnt, such as Akt/mTOR pathway components (e.g., S6K and 4E-BP1) in rat pheochromocytoma cells (Schmitz et al. 2019). Importantly, GSK3β is a protein that plays a role in both pathways and can be inactivated by dopamine-mediated inactivation of Akt (Beaulieu et al. 2007). Moreover, the Wnt signaling seems to be strongly related to the dopaminergic system, which can be modulated by MPH due to its function as a DAT blocker (Han et al. 2019). In their article, Han and colleagues discussed how dopamine D2 receptor-mediated Wnt activation regulated kidney cell proliferation (Han et al. 2019). Similarly, another piece of evidence has demonstrated that the hippocampal neurogenesis of murine models of Parkinson’s Disease was increased after the Wnt signaling was activated in a process mediated by the dopamine D1 receptor (Mishra et al. 2019).

On the other hand, unlike ADHD NSCs, the administration of DKK1 only reduced the proliferation of control NSCs in our findings. Given that previous studies have already reported that inhibiting the Wnt signaling in other cell lines (such as in the context of cancer) leads to deficits in proliferation (Martinez-Font et al. 2020; Bilir et al. 2013), this finding was to be expected. Regarding ADHD NSCs, we hypothesize that group-specific cellular responses might lead to unaltered proliferation, such as a potential compensatory mechanism triggered by molecular dysregulations through other pathways that crosstalk with Wnt and regulate proliferation (Lee et al. 2021a).

Our study found that increased levels of active β-catenin were associated with diminished NSC proliferation, suggesting that elevated basal Wnt activity observed in ADHD NSCs may be a compensatory mechanism to boost proliferation (Walter et al. 2024). However, the interactions between Wnt pathway and other pathways may also explain this. Since chronic MPH at 10 nM increases NSC proliferation in ADHD patients, it may improve compensatory efforts.

We acknowledge that some of this paper’s techniques may be limited. Western Blots may introduce a large variability. As qualitative protein expression assessments, normalization and reproducibility may be challenging. Automated assays and ELISA are more sensitive. Furthermore, this methodology only depicts the exact time frame of protein extraction after chronic MPH treatment, not Wnt functionality, even though we examined three proteins at distinct cascade points.

The relatively short duration of MPH treatment in proliferation and Western Blot experiments may also explain why ADHD NSC proliferation does not fully recover and the expression of Wnt proteins remains unaltered after treatment. This context may not accurately reflect patient treatment, but it indicates the ideal duration for *in vitro* treatment to yield more reliable results.

The sample size used in this study may affect the results and explain certain experimental tendencies (Dutan Polit et al. 2023; Beekhuis-Hoekstra et al. 2021). While generating iPSC lines is expensive and time-consuming, increasing the sample size of patients and controls would help determine whether these trends become more pronounced and statistically significant. MPH’s effects must be studied in detail to understand its mechanisms. This research is ongoing in our laboratory. This study suggests that MPH targets the Wnt pathway and links it to ADHD’s proliferative phenotype using patient-specific cell lines. Although the results are tentative, this is the first publication to do so.

Nevertheless, our findings suggest the need for further research into novel MPH targets, including Wnt and other pathways in ADHD and ADHD-related phenotypes. Wnt signaling regulates cell proliferation, maturation, and differentiation; therefore, ADHD disease modeling should examine differences in neuronal or glial differentiation and functionality between ADHD patients and controls. This will help gain a more comprehensive understanding of the precise role played by the canonical Wnt signaling pathway in these circumstances.

## Supplementary Information

Below is the link to the electronic supplementary material.

Supplementary file1 (PDF 2462 kb)

Supplementary file2 (XLSX 30 kb)

Supplementary file3 (XLSX 28 kb)

## Data Availability

Data and R codes used for the calculation of proliferation rates of xCELLigence, the generation of the correlation matrices, and all statistical analyses in this study can be provided upon request.
