# Supplementary material for: Assessing the effects of methylphenidate in proliferation and Wnt activity of neuronal stem cells from attention deficit/hyperactivity disorder patients
Source: J Neural Transm (Vienna). 2025 Jul 28;133(1):169–88. doi: 10.1007/s00702-025-02988-y (PMC12827369; doi:10.1007/s00702-025-02988-y)
Supplement: Supplementary file 1 — Supplementary file1 (PDF 2462 kb) [file 702_2025_2988_MOESM1_ESM.pdf]

Assessing the effects of methylphenidate in proliferation and Wnt activity of neuronal stem cells from Attention Deficit/Hyperactivity Disorder patients

Cristine Marie Yde Ohki, Ph.D.[1], Natalie Monet Walter, M.Sc.[1], Lukasz Smigielski, Ph.D.[1], Audrey Bender, M.Sc.[1], Michelle Rickli, M.Sc.[1], Susanne Walitza, Prof. Dr. MD MSc[1,2,3], Edna Grünblatt, Prof. Ph.D.[*1,2,3]

[1] Department of Child and Adolescent Psychiatry and Psychotherapy, Translational Molecular Psychiatry, University Hospital of Psychiatry Zurich, University of Zurich, Wagistrasse 12, 8952, Schlieren, Switzerland

[2] Neuroscience Center Zurich, University of Zurich and the ETH Zurich, Winterthurerstrasse 11, 8057, Zurich, Switzerland

[3] Zurich Center for Integrative Human Physiology, University of Zurich, Winterthurerstrasse 11, 8057, Zurich, Switzerland

[*]Correspondence:

Edna Grünblatt

Head of Translational Molecular Psychiatry

Chair of ECNP TWG iPSC Platform for Neuropsychiatry

Department of Child and Adolescent Psychiatry and Psychotherapy

University Hospital of Psychiatry Zurich, University of Zurich (UZH)

Wagistrasse 12; CH-8952 Schlieren/ Switzerland

E-mail: edna.gruenblatt@kjpd.uzh.ch

This file contains Supplementary Figures 1 – 6 and Supplementary Tables 1 - 5 from the manuscript from Yde Ohki et al. Supplementary Tables 6 and 7 are individually attached.

Supplementary Information

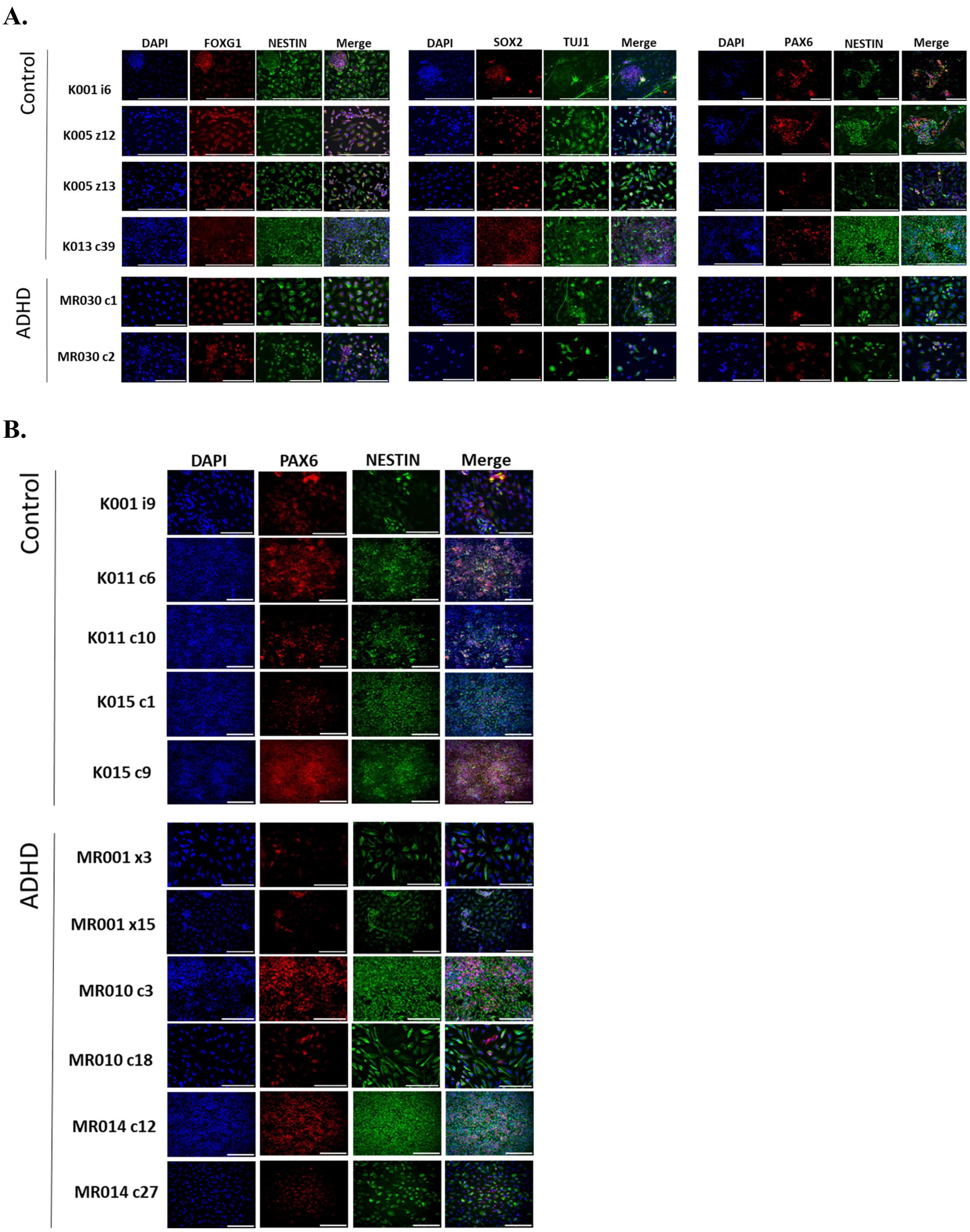

Supplementary Fig. 1: Immunocytochemistry images for quality control of iPSC-derived NSCs from ADHD and controls. A) Complementary staining to the QC reported in Yde Ohki et al. 2023a, showing the protein expression of Nestin and PAX6 in NSCs K001 i9, K011 c6/c10, K015 c1/c9, MR001 x1/x15, MR010 c3/c10 and MR014 c12/c27 (Supplementary Table 2). Scale bar: 200 µm. B) Expression of PAX6, NESTIN, SOX2, FOXG1 and TUJ1 in the remaining NSCs (Supplementary Table 2). Scale bar: 200 µm, with exception for NSCs K001 i9, MR001 x3 and MR010 c3/c18 (scale bar: 100 µm).

A.

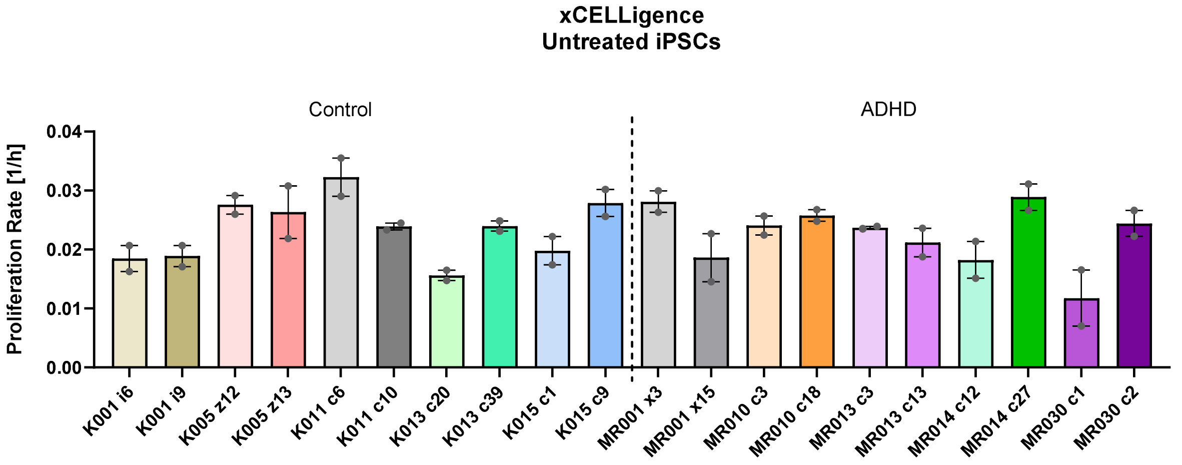

B.

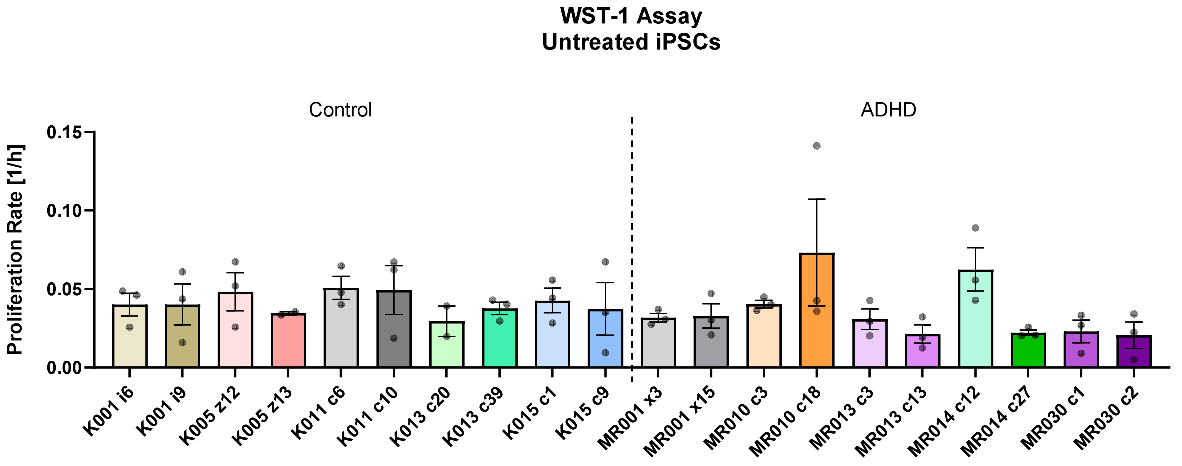

Supplementary Fig. 2: Proliferation rates from all iPSC lines included in this study at baseline. Proliferation rates derived from xCELLigence experiments (A) and WST-1 assays (B) are shown in the graphs. Mean ± SEM for each cell line is depicted, while each dot represents one technical replicate. At least 2 technical replicates were considered to calculate individual means in this analysis.

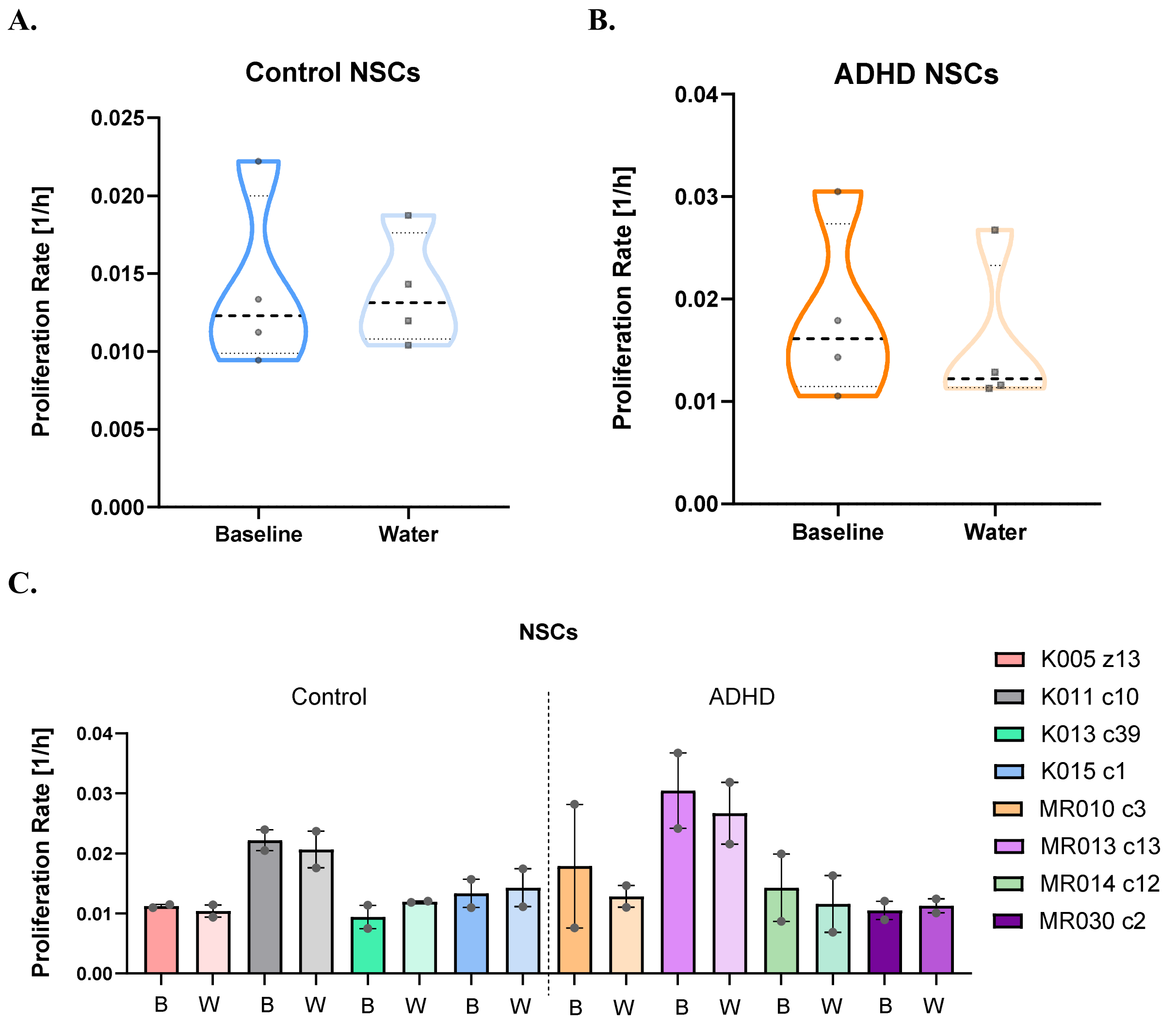

Supplementary Fig. 3: Proliferation rates from NSC lines cultured as baseline or with water as vehicle treatment. Proliferation rates derived from xCELLigence experiments in control (A) and ADHD (B) NSCs are shown in the graphs. For both groups, no differences are seen between the conditions (Mann-Whitney, n.s.). C) Proliferation rates from individual cell lines in these two conditions. B= Baseline condition; W = Water condition. Mean ± SEM for each cell line is depicted, while each dot represents one technical replicate. N=4 Control individuals (1 clone each) and 4 ADHD patients (1 clone each) were analyzed in 2 technical replicates.

A.

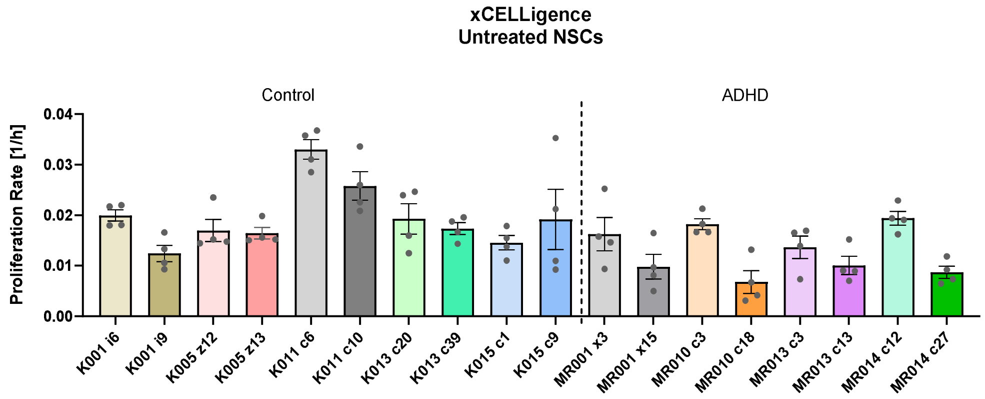

B.

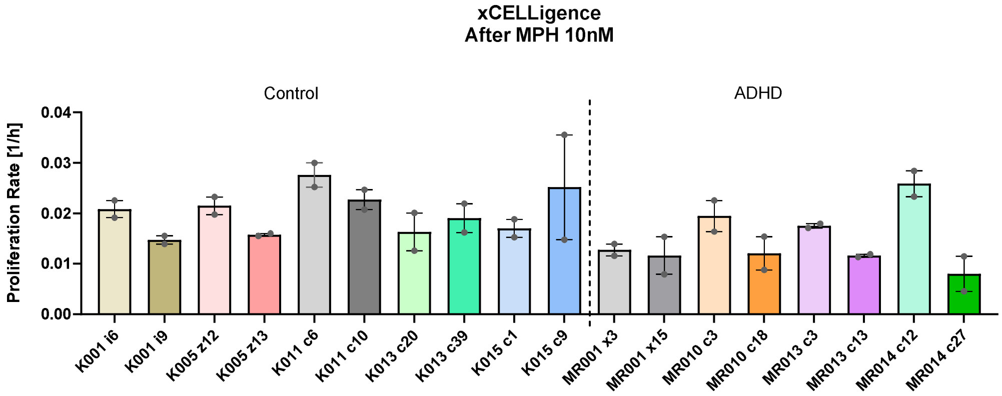

C.

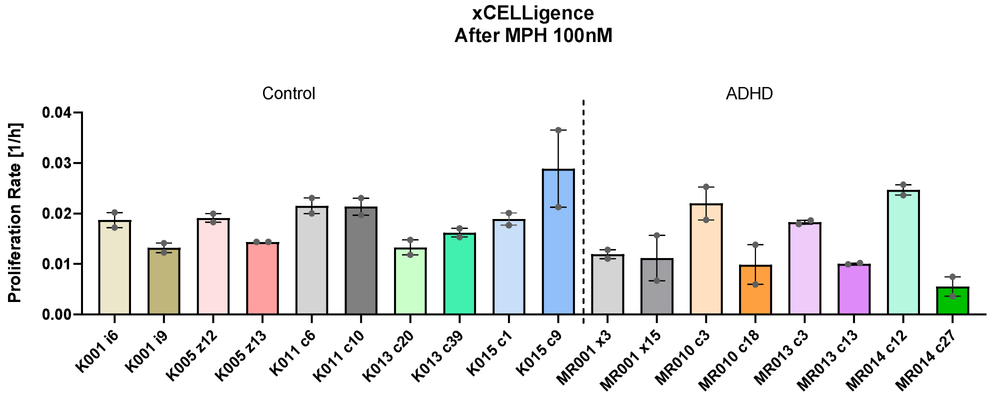

Supplementary Fig. 4: Proliferation rates of all the NSC lines analyzed in xCELLigence. The graphs depict proliferation rates for individual NSC lines at baseline (A) and after chronic treatment with MPH 10 nM (B) and 100 nM (C). For the vehicle condition, four experiments per cell line were considered. Mean ± SEM for each cell line is depicted, while each dot represents one technical replicate. N=2 technical replicates were performed for each cell line.

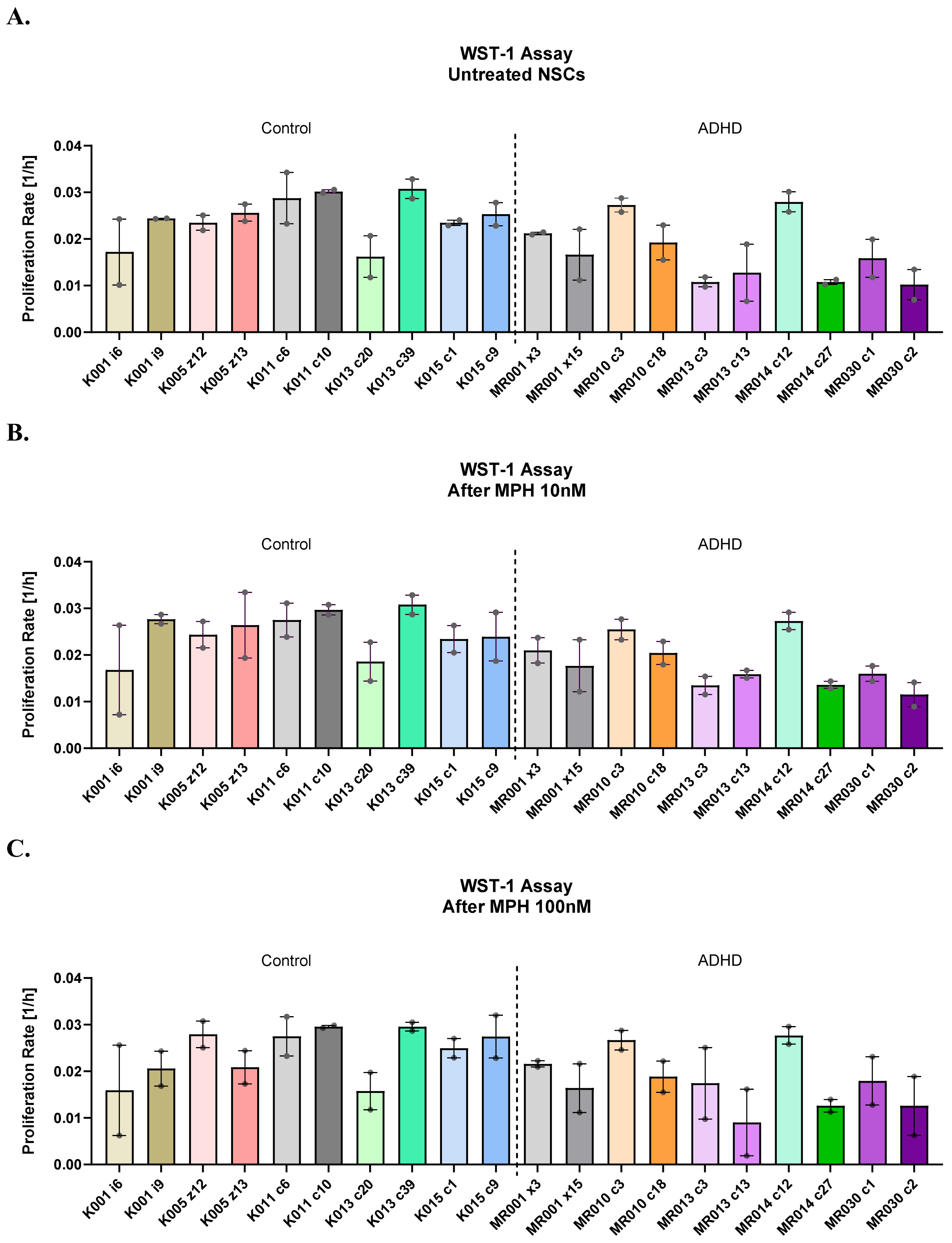

Supplementary Fig. 5: Proliferation rates of all the NSC lines analyzed in WST-1 assays. The graphs depict proliferation rates for individual NSC lines at baseline (A) and after chronic treatment with MPH 10nM (B) and 100nM (C). Mean ± SEM for each cell line is depicted, while each dot represents one technical replicate. N=2 technical replicates were performed for each cell line.

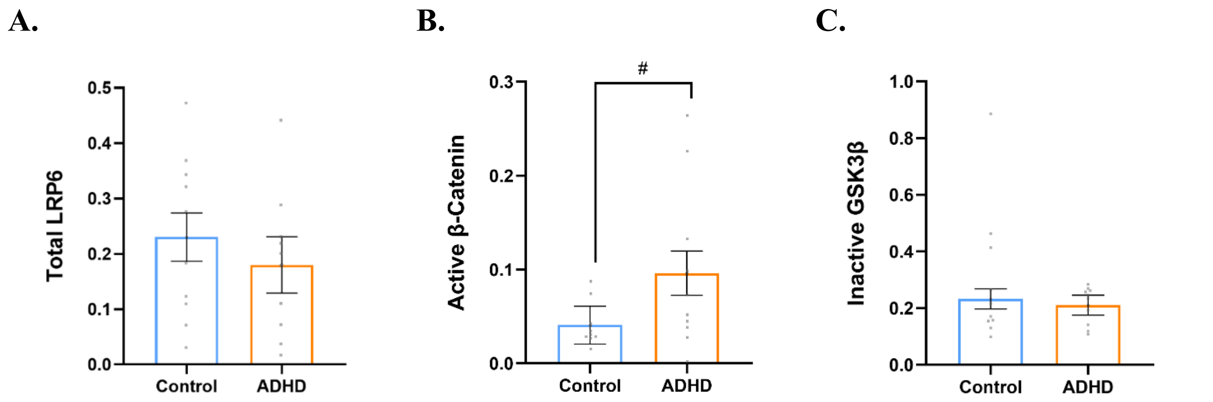

Supplementary Fig. 6: Protein expression of Wnt-related proteins between control and ADHD NSCs at baseline after 7-day culture: Total LRP6 (A), active β-catenin (B) and inactive GSK3β (C). No statistical differences were seen for any of the proteins. A trend toward significance was observed for active β-catenin ($^{\#}p = 0.068$). Mean ± SEM (model-adjusted, standard *lmer* for total LRP6 and active β-catenin; robust *lmer* for inactive GSK3β) is depicted, while each dot represents the averaged raw data from 2 experiments per cell line. N=2 technical replicates were performed for each cell line.

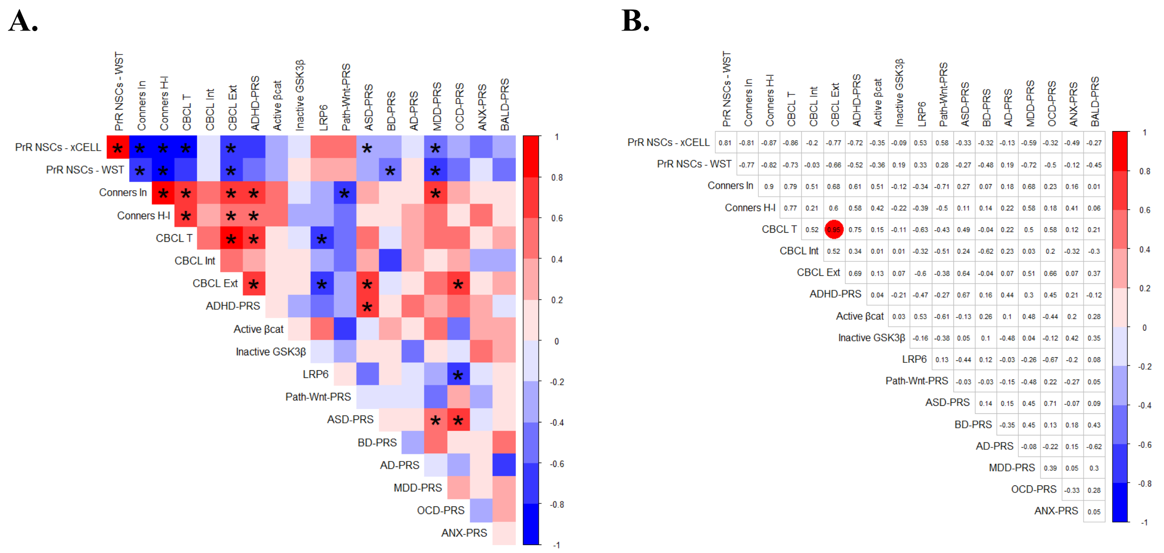

Supplementary Fig. 7: Correlations between ADHD-related behavioral scores, Wnt-PRS, PRS for ADHD and other disorders and *in vitro* findings when results from two iPSC clones were averaged. A) Uncorrected pairwise comparisons after averaging *in vitro* findings of two clones derived from the same donor. As in Fig. 5A, Spearman's correlations were conducted in this analysis while colors code the strength and direction of each correlation. Asterisks indicate statistically significant correlations without any corrections applied ($p < 0.05$). B) Correlation coefficients after Bonferroni corrections are shown. After a conservative Bonferroni's correction for 171 unique pair combinations, 1 pair survived the correction (0.05/171, in red, $p < 0.05$): CBCL T *versus* CBCL Ext. The red color indicate the positive correlation whereas 0.95 represents the correlation coefficient. Abbreviations: PrR NSCs – xCELL: Proliferation rates of NSCs from xCELLigence assays; PrR NSCs – WST: Proliferation rates of NSCs from WST-1 assays; Conners In = Inattention scores from Conners' Rating Scales; Conners H-I: Hyperactivity/Impulsivity scores from Conners' Rating Scales; CBCL T = Total Scores from CBCL; CBCL Int = Internalizing scores from CBCL; CBCL Ext = Externalizing scores from CBCL; Active βcat = Protein expression of active β-catenin after a 7-day cell culture; Inactive GSK3β = Protein expression of inactive GSK3β after a 7-day cell culture; LRP6 = Protein expression of LRP6 after a 7-day cell culture; ADHD-PRS = Polygenic Risk Scores for Attention-Deficit Hyperactivity Disorder; Path-Wnt-PRS = Pathway-PRS specific to Wnt signaling; ASD-PRS = Polygenic Risk Scores for Autism Spectrum Disorder; BD-PRS = Polygenic Risk Scores for Bipolar Disorder; AD-PRS = Polygenic Risk Scores for Alzheimer's Disease; MDD-PRS = Polygenic Risk Scores for Major Depressive Disorder; OCD-PRS = Polygenic Risk Scores for Obsessive-Compulsive Disorder; ANX-PRS = Polygenic Risk Scores for Anxiety Disorder. Genetic variables and clinical scores were also present in a previous publication (Walter et al. 2024).

Supplementary Table 1: Demographic and clinical information about subjects analyzed in this study.

Cell line	Clones analyzed in this study	Cell type of origin	Reprogramming method	Diagnosis	Ethnicity	Age	Sex	ADHD-PRS (z-score)	IQ	Conners		CBCL		
										I	H/I	Total	Ext	Int
K001	i6 & i9	Keratinocytes	Sendai Virus transduction	Healthy control	Caucasian	15	Male	0.46	118	57	50	54	51	55
K005	z12 & z13	Keratinocytes	Sendai Virus transduction	Healthy control	Caucasian	16	Male	-0.52	113	53	48	48	45	53
K011	c6 & c10	Keratinocytes	Sendai Virus transduction	Healthy control	Caucasian	16	Male	-1.03	124	39	48	43	34	51
K013	c20 & c39	PBMCs	Sendai Virus transduction	Healthy control	Caucasian	9	Female	-0.08	127	43	47	52	53	53
K015	c1 & c9	PBMCs	Sendai Virus transduction	Healthy control	Caucasian	13	Male	-1.66	127	30	38	39	40	49
MR001	x3 & x15	Keratinocytes	Sendai Virus transduction	ADHD	Caucasian	15	Male	-0.67	121	73	69	63	60	61
MR010	c3 & c18	Keratinocytes	Sendai Virus transduction	ADHD	Caucasian	9	Male	1.63	133	73	69	63	60	61
MR013	c3 & c13	PBMCs	Sendai Virus transduction	ADHD	Caucasian	16	Male	0.29	109	73	73	53	50	49
MR014	c12 & c27	PBMCs	Sendai Virus transduction	ADHD	Caucasian	13	Male	0.45	98	73	58	59	57	57
MR030	c1 & c2	PBMCs	Sendai Virus transduction	ADHD	Caucasian	9	Female	1.13	143	66	69	68	74	50

Individual ADHD-PRS were calculated using Demontis et al. 2023[31] and a threshold of p=0.05 was adopted. T-values from Conners' Rating Scales and Child Behavior Checklist (CBCL) scores are represented. Abbreviations: I = Inattention; H/I = Hyperactivity/Impulsivity; Total = Total scale scores; Ext = Externalizing scale scores; Int = Internalizing scale scores.

Supplementary Table 2: Location of QC information from iPSCs and iPSC-derived NSCs.

Donor	Clone	QC of iPSCs	QC of NSCs	
		Gene/protein expression, EB formation, genomic integrity, detection of mycoplasma/Sendai virus & CNV analysis reported on:	Gene expression via RT-PCR reported on:	Protein expression via Immunocytochemistry reported on:
K001	i6	Yde Ohki et al. 2021	Walter et al. 2024	Suppl. Fig. 1
	i9	Yde Ohki et al. 2021	Yde Ohki et al. 2023a & Walter et al. 2024	Yde Ohki et al. 2023a & Suppl. Fig. 1
K005	z12	Yde Ohki et al. 2021	Walter et al. 2024	Suppl. Fig. 1
	z13	Yde Ohki et al. 2021	Walter et al. 2024	Suppl. Fig. 1
K011	c6	Yde Ohki et al. 2021	Yde Ohki et al. 2023a & Walter et al. 2024	Yde Ohki et al. 2023a & Suppl. Fig. 1
	c10	Yde Ohki et al. 2021	Yde Ohki et al. 2023a & Walter et al. 2024	Yde Ohki et al. 2023a & Suppl. Fig. 1
K013	c20	Yde Ohki et al. 2023b	Walter et al. 2024	Walter et al. 2024
	c39	Yde Ohki et al. 2023b	Walter et al. 2024	Suppl. Fig. 1
K015	c1	Yde Ohki et al. 2023b	Yde Ohki et al. 2023a & Walter et al. 2024	Yde Ohki et al. 2023a & Suppl. Fig. 1
	c9	Yde Ohki et al. 2023b	Yde Ohki et al. 2023a & Walter et al. 2024	Yde Ohki et al. 2023a & Suppl. Fig. 1
MR001	x3	Grossmann et al. 2021	Yde Ohki et al. 2023a & Walter et al. 2024	Yde Ohki et al. 2023a & Suppl. Fig. 1
	x15	Grossmann et al. 2021	Yde Ohki et al. 2023a & Walter et al. 2024	Yde Ohki et al. 2023a & Suppl. Fig. 1
MR010	c3	Grossmann et al. 2021	Yde Ohki et al. 2023a & Walter et al. 2024	Yde Ohki et al. 2023a & Suppl. Fig. 1
	c18	Grossmann et al. 2021	Yde Ohki et al. 2023a & Walter et al. 2024	Yde Ohki et al. 2023a & Suppl. Fig. 1
MR013	c3	Grossmann et al. 2021	Walter et al. 2024	Walter et al. 2024 & Suppl. Fig. 1
	c13	Yde Ohki et al. 2023b	Walter et al. 2024	Walter et al. 2024
MR014	c12	Grossmann et al. 2021	Yde Ohki et al. 2023a & Walter et al. 2024	Yde Ohki et al. 2023a & Suppl. Fig. 1
	c27	Grossmann et al. 2021	Yde Ohki et al. 2023a & Walter et al. 2024	Yde Ohki et al. 2023a & Suppl. Fig. 1
MR030	c1	Yde Ohki et al. 2024	Walter et al. 2024	Suppl. Fig. 1
	c2	Yde Ohki et al. 2024	Walter et al. 2024	Suppl. Fig. 1

Supplementary Table 3: Primary and secondary antibodies for Western Blot

Type of Antibody	Primary Antibodies	Product	Company	Species	Dilution	Size
Primary Antibody	Anti-β-Catenin non-phosphorylated, active	D13A1	Cell Signaling Technology	Rabbit	1: 1000	92 kDa
	Anti-GSK3β	ab32391	Abcam	Rabbit	1: 5000	46 kDa
	Anti-GSK3β Phospho S9	ab75814	Abcam	Rabbit	1: 5000	47 kDa
	Anti-LRP6	ab134146	Abcam	Rabbit	1: 500	180 kDa
	Anti-GAPDH loading control	ab8245	Abcam	Mouse	1: 2000	40 kDa
Secondary Antibody	Goat Anti-Rabbit IgG (HRP)	ab7090	Abcam	Goat	1:10000	N/A
	Donkey Anti-Mouse IgG(HRP)	ab7061	Abcam	Donkey	1:10000	N/A

Supplementary Table 4: Summary of all experiments conducted in NSC lines included in this study.

Donor	Clone	Experiment								
		**Baseline x Vehicle Comparison (xCELLigence)**	**Basal proliferation of NSCs (xCELLigence)**	**Basal proliferation of NSCs (WST-1)**	**Proliferation of NSCs after MPH and/or DKK1 (xCELLigence)**	**Proliferation of NSCs after MPH (WST-1)**	**Basal Wnt-protein expression (Western Blot)**	**Wnt-protein expression before and after MPH (Western Blot)**	**Basal Wnt activity (Wnt reporter assays)**	**Wnt activity after MPH (Wnt reporter assays)**
K001	i6	N/A	✓✓ [Fig. 1 & Suppl. Fig. 4]	✓✓ [Fig. 1]	✓✓ [Figs. 2B & 3; Suppl. Fig. 4]	✓✓ [Fig. 2C; Suppl. Fig. 5]	✓✓ [Walter et al. 2024]	✓✓ [Fig. 4 & Suppl. Fig. 6]	✓✓ [Walter et al. 2024]	✓✓ [Fig. 4F]
	i9	N/A	✓✓ [Fig. 1 & Yde Ohki et al. 2023a]	✓✓ [Fig. 1 & Yde Ohki et al. 2023a]	✓✓ [Figs. 2B & 3; Suppl. Fig. 4]	✓✓ [Fig. 2C; Suppl. Fig. 5]	✓✓ [Walter et al. 2024]	✓✓ [Fig. 4 & Suppl. Fig. 6]	✓✓ [Walter et al. 2024]	✓✓ [Fig. 4F]
K005	z12	N/A	✓✓ [Fig. 1]	✓✓ [Fig. 1]	✓✓ [Figs. 2B & 3; Suppl. Fig. 4]	✓✓ [Fig. 2C; Suppl. Fig. 5]	✓✓ [Walter et al. 2024]	✓✓ [Fig. 4 & Suppl. Fig. 6]	✓✓ [Walter et al. 2024]	✓✓ [Fig. 4F]
	z13	✓✓ [Suppl. Fig. 3]	✓✓ [Fig. 1]	✓✓ [Fig. 1]	✓✓ [Figs. 2B & 3; Suppl. Fig. 4]	✓✓ [Fig. 2C; Suppl. Fig. 5]	✓✓ [Walter et al. 2024]	✓✓ [Fig. 4 & Suppl. Fig. 6]	✓✓ [Walter et al. 2024]	✓✓ [Fig. 4F]
K011	c6	N/A	✓✓ [Fig. 1 & Yde Ohki et al. 2023a]	✓✓ [Fig. 1 & Yde Ohki et al. 2023a]	✓✓ [Figs. 2B & 3; Suppl. Fig. 4]	✓✓ [Fig. 2C; Suppl. Fig. 5]	✓✓ [Walter et al. 2024]	✓✓ [Fig. 4 & Suppl. Fig. 6]	✓✓ [Walter et al. 2024]	✓✓ [Fig. 4F]
	c10	✓✓ [Suppl. Fig. 3]	✓✓ [Fig. 1 & Yde Ohki et al. 2023a]	✓✓ [Fig. 1 & Yde Ohki et al. 2023a]	✓✓ [Figs. 2B & 3; Suppl. Fig. 4]	✓✓ [Fig. 2C; Suppl. Fig. 5]	✓✓ [Walter et al. 2024]	✓✓ [Fig. 4 & Suppl. Fig. 6]	✓✓ [Walter et al. 2024]	✓✓ [Fig. 4F]
K013	c20	N/A	✓✓ [Fig. 1]	✓✓ [Fig. 1]	✓✓ [Figs. 2B & 3; Suppl. Fig. 4]	✓✓ [Fig. 2C; Suppl. Fig. 5]	✓✓ [Walter et al. 2024]	✓✓ [Fig. 4 & Suppl. Fig. 6]	✓✓ [Walter et al. 2024]	✓✓ [Fig. 4F]
	c39	✓✓ [Suppl. Fig. 3]	✓✓ [Fig. 1]	✓✓ [Fig. 1]	✓✓ [Figs. 2B & 3; Suppl. Fig. 4]	✓✓ [Fig. 2C; Suppl. Fig. 5]	✓✓ [Walter et al. 2024]	✓✓ [Fig. 4 & Suppl. Fig. 6]	✓✓ [Walter et al. 2024]	✓✓ [Fig. 4F]
K015	c1	✓✓ [Suppl. Fig. 3]	✓✓ [Fig. 1 & Yde Ohki et al. 2023a]	✓✓ [Fig. 1 & Yde Ohki et al. 2023a]	✓✓ [Figs. 2B & 3; Suppl. Fig. 4]	✓✓ [Fig. 2C; Suppl. Fig. 5]	✓✓ [Walter et al. 2024]	✓✓ [Fig. 4 & Suppl. Fig. 6]	✓✓ [Walter et al. 2024]	✓✓ [Fig. 4F]
	c9	N/A	✓✓ [Fig. 1 & Yde Ohki et al. 2023a]	✓✓ [Fig. 1 & Yde Ohki et al. 2023a]	✓✓ [Figs. 2B & 3; Suppl. Fig. 4]	✓✓ [Fig. 2C; Suppl. Fig. 5]	✓✓ [Walter et al. 2024]	✓✓ [Fig. 4 & Suppl. Fig. 6]	✓✓ [Walter et al. 2024]	✓✓ [Fig. 4F]
MR001	x3	N/A	✓✓ [Fig. 1 & Yde Ohki et al. 2023a]	✓✓ [Fig. 1 & Yde Ohki et al. 2023a]	✓✓ [Figs. 2B & 3; Suppl. Fig. 4]	✓✓ [Fig. 2C; Suppl. Fig. 5]	✓✓ [Walter et al. 2024]	✓✓ [Fig. 4 & Suppl. Fig. 6]	✓✓ [Walter et al. 2024]	✓✓ [Fig. 4F]
	x15	N/A	✓✓ [Fig. 1 & Yde Ohki et al. 2023a]	✓✓ [Fig. 1 & Yde Ohki et al. 2023a]	✓✓ [Figs. 2B & 3; Suppl. Fig. 4]	✓✓ [Fig. 2C; Suppl. Fig. 5]	✓✓ [Walter et al. 2024]	✓✓ [Fig. 4 & Suppl. Fig. 6]	✓✓ [Walter et al. 2024]	✓✓ [Fig. 4F]
MR010	c3	✓✓ [Suppl. Fig. 3]	✓✓ [Fig. 1 & Yde Ohki et al. 2023a]	✓✓ [Fig. 1 & Yde Ohki et al. 2023a]	✓✓ [Figs. 2B & 3; Suppl. Fig. 4]	✓✓ [Fig. 2C; Suppl. Fig. 5]	✓✓ [Walter et al. 2024]	✓✓ [Fig. 4 & Suppl. Fig. 6]	✓✓ [Walter et al. 2024]	✓✓ [Fig. 4F]
	c18	N/A	✓✓ [Fig. 1 & Yde Ohki et al. 2023a]	✓✓ [Fig. 1 & Yde Ohki et al. 2023a]	✓✓ [Figs. 2B & 3; Suppl. Fig. 4]	✓✓ [Fig. 2C; Suppl. Fig. 5]	✓✓ [Walter et al. 2024]	✓✓ [Fig. 4 & Suppl. Fig. 6]	✓✓ [Walter et al. 2024]	✓✓ [Fig. 4F]
MR013	c3	N/A	✓✓ [Fig. 1]	✓✓ [Fig. 1]	✓✓ [Figs. 2B & 3; Suppl. Fig. 4]	✓✓ [Fig. 2C; Suppl. Fig. 5]	✓✓ [Walter et al. 2024]	✓✓ [Fig. 4 & Suppl. Fig. 6]	✓✓ [Walter et al. 2024]	✓✓ [Fig. 4F]
	c13	✓✓ [Suppl. Fig. 3]	✓✓ [Fig. 1]	✓✓ [Fig. 1]	✓✓ [Figs. 2B & 3; Suppl. Fig. 4]	✓✓ [Fig. 2C; Suppl. Fig. 5]	✓✓ [Walter et al. 2024]	✓✓ [Fig. 4 & Suppl. Fig. 6]	✓✓ [Walter et al. 2024]	✓✓ [Fig. 4F]
MR014	c12	✓✓ [Suppl. Fig. 3]	✓✓ [Fig. 1 & Yde Ohki et al. 2023a]	✓✓ [Fig. 1 & Yde Ohki et al. 2023a]	✓✓ [Figs. 2B & 3; Suppl. Fig. 4]	✓✓ [Fig. 2C; Suppl. Fig. 5]	✓✓ [Walter et al. 2024]	✓✓ [Fig. 4 & Suppl. Fig. 6]	✓✓ [Walter et al. 2024]	✓✓ [Fig. 4F]
	c27	N/A	✓✓ [Fig. 1 & Yde Ohki et al. 2023a]	✓✓ [Fig. 1 & Yde Ohki et al. 2023a]	✓✓ [Figs. 2B & 3; Suppl. Fig. 4]	✓✓ [Fig. 2C; Suppl. Fig. 5]	✓✓ [Walter et al. 2024]	✓✓ [Fig. 4 & Suppl. Fig. 6]	✓✓ [Walter et al. 2024]	✓✓ [Fig. 4F]
MR030	c1	N/A	✓✓ [Fig. 1]	✓✓ [Fig. 1]	✓✓ [Figs. 2B & 3; Suppl. Fig. 4]	✓✓ [Fig. 2C; Suppl. Fig. 5]	✓✓ [Walter et al. 2024]	✓✓ [Fig. 4 & Suppl. Fig. 6]	✓✓ [Walter et al. 2024]	✓✓ [Fig. 4F]
	c2	✓✓ [Suppl. Fig. 3]	✓✓ [Fig. 1]	✓✓ [Fig. 1]	✓✓ [Figs. 2B & 3; Suppl. Fig. 4]	✓✓ [Fig. 2C; Suppl. Fig. 5]	✓✓ [Walter et al. 2024]	✓✓ [Fig. 4 & Suppl. Fig. 6]	✓✓ [Walter et al. 2024]	✓✓ [Fig. 4F]

Each independent experiment is represented by one check mark (✓). N/A = not applicable.

Supplementary Table 5: Statistics from the *posthoc* tests applied in this study.

Experiment	Figure	Comparison	Est.	SE	t- or z-ratio	df	p-value
NSC Proliferation after MPH treatment using xCELLigence assays	2B	ADHD Vehicle *versus* Control Vehicle	-0.37	0.13	-2.89	14.00	**0.012**
		ADHD MPH 10 nM *versus* Control MPH 10 nM	-0.29	0.13	-2.23	14.00	**0.043**
		ADHD MPH 100 nM *versus* Control MPH 100 nM	-0.27	0.13	-2.10	14.00	*0.055*
NSC Proliferation after MPH treatment using WST-1 assays	2C	ADHD Vehicle *versus* Control Vehicle	-0.30	0.11	-2.80	9.54	**0.020**
		ADHD MPH 10 nM *versus* Control MPH 10 nM	-0.27	0.11	-2.57	9.54	**0.029**
		ADHD MPH 100 nM *versus* Control MPH 100 nM	-0.24	0.11	-2.29	9.54	**0.046**
NSC Proliferation after MPH and/or DKK1 treatments using xCELLigence assays	3B	ADHD Vehicle *versus* Control Vehicle	-0.36	0.17	-2.16	12.60	*0.051*
		ADHD MPH 10 nM *versus* Control MPH 10 nM	-0.28	0.17	-1.65	12.60	0.124
		ADHD DKK1 *versus* Control DKK1	-0.29	0.17	0.11	12.60	0.110
		ADHD DKK1 + MPH *versus* Control DKK1 + MPH	-0.33	0.17	-1.96	12.60	*0.073*

Statistically significant p-values ($p < 0.05$) are highlighted in **bold**, while trends toward significance ($0.05 < p < 0.075$) are written in *italic*.

Supplementary Table 6: Pair of all correlations considering clones individually (N=20), as well as their respective correlation coefficients and p-values.

See file attached as supplementary material.

Supplementary Table 7: Pair of all correlations after averaging results from clones (N=10), as well as their respective correlation coefficients and p-values.

See file attached as supplementary material.

References

Grossmann L, Yde Ohki CM, Doring C, Hoffmann P, Herms S, Werling AM, Walitza S, Grünblatt E (2021) Generation of integration-free induced pluripotent stem cell lines from four pediatric ADHD patients. Stem Cell Res 53: 102268

Walter NM, Yde Ohki CM, Rickli M, Smigielski L, Walitza S, Grünblatt E (2024) An investigation on the alterations in Wnt signaling in ADHD across developmental stages. Neuroscience Applied 3: 104070

Yde Ohki CM, Grossmann L, Doring C, Hoffmann P, Herms S, Werling AM, Walitza S, Grünblatt E (2021) Generation of integration-free induced pluripotent stem cells from healthy individuals. Stem Cell Res 53: 102269

Yde Ohki CM, Walter NM, Bender A, Rickli M, Ruhstaller S, Walitza S, Grünblatt E (2023a) Growth rates of human induced pluripotent stem cells and neural stem cells from attention-deficit hyperactivity disorder patients: a preliminary study. J Neural Transm (Vienna) 130(3): 243–252

Yde Ohki CM, Walter NM, Rickli M, Iseli C, Werling AM, Döring C, Rubio B, Hoffmann P, Herms S, Walitza S, Grünblatt E (2024) Generation of induced pluripotent stem cells from ADHD patients who do or do not respond to Methylphenidate treatment. Stem Cell Res 81: 103546

Yde Ohki CM, Walter NM, Rickli M, van Puyenbroeck P, Doring C, Hoffmann P, Herms S, Werling AM, Walitza S, Grünblatt E (2023b) Generation of induced pluripotent stem cells from two ADHD patients and two healthy controls. Stem Cell Res 69: 103084
